# Novel Regulatory Small RNAs in *Streptococcus pyogenes*

**DOI:** 10.1371/journal.pone.0064021

**Published:** 2013-06-06

**Authors:** Rafael A. Tesorero, Ning Yu, Jordan O. Wright, Juan P. Svencionis, Qiang Cheng, Jeong-Ho Kim, Kyu Hong Cho

**Affiliations:** 1 Department of Microbiology, Southern Illinois University, Carbondale, Illinois, United States of America; 2 Department of Computer Science, Southern Illinois University, Carbondale, Illinois, United States of America; 3 Department of Biochemistry and Molecular Biology, The George Washington University Medical Center, Washington, District of Columbia, United States of America; Centers for Disease Control & Prevention, United States of America

## Abstract

*Streptococcus pyogenes* (Group A *Streptococcus* or GAS) is a Gram-positive bacterial pathogen that has shown complex modes of regulation of its virulence factors to cause diverse diseases. Bacterial small RNAs are regarded as novel widespread regulators of gene expression in response to environmental signals. Recent studies have revealed that several small RNAs (sRNAs) have an important role in *S. pyogenes* physiology and pathogenesis by regulating gene expression at the translational level. To search for new sRNAs in *S. pyogenes*, we performed a genomewide analysis through computational prediction followed by experimental verification. To overcome the limitation of low accuracy in computational prediction, we employed a combination of three different computational algorithms (sRNAPredict, eQRNA and RNAz). A total of 45 candidates were chosen based on the computational analysis, and their transcription was analyzed by reverse-transcriptase PCR and Northern blot. Through this process, we discovered 7 putative novel *trans*-acting sRNAs. Their abundance varied between different growth phases, suggesting that their expression is influenced by environmental or internal signals. Further, to screen target mRNAs of an sRNA, we employed differential RNA sequencing analysis. This study provides a significant resource for future study of small RNAs and their roles in physiology and pathogenesis of *S. pyogenes*.

## Introduction

Small non-coding RNAs exist in all life forms and are now regarded as novel widespread regulators of gene expression. In bacteria, these RNAs are collectively referred to as ‘small RNAs’ or ‘sRNAs’. Bacterial small regulatory RNAs recently have received tremendous attention because of their abundance and important role in a variety of cellular processes including response to environmental stress and involvement in pathogenecity (for recent reviews, refer to [1], [2], [3], [4], [5], [6]). While bacterial sRNAs show dramatic heterogeneity in size (30–500 nucleotides in length) and structure, their functional roles are similar to each other; they regulate gene expression mostly at the translational level in response to environmental signals [4], [7]. Since regulation at the translational level results in a quicker effect than that at the transcriptional level, bacteria use sRNAs for immediate response to environmental change, growth phase, and immune reaction. Most sRNAs participate in post-transcriptional regulation by base-pairing with target mRNAs, which results in the regulation (both inhibition or activation) of translation or degradation of the mRNAs [8]. A small fraction of sRNAs interact with RNA-binding proteins to modify their activities [9]. The sRNAs that bind to target mRNAs often have 5′ and 3′ stem-loop structures flanking central unpaired regions, while the sRNAs that bind to proteins other than RNA chaperones often fold into highly paired, extended hairpin structures [9].

*S. pyogenes* is a Gram-positive pathogen, which causes diseases ranging from mild superficial infections such as pharyngitis and impetigo to life-threatening systemic diseases including toxic shock and necrotizing fasciitis. These diseases still remain a major public health concern both in developed and developing countries. More than 30 million cases of streptococcal pharyngitis occur each year in the USA. Worldwide, *S. pyogenes* causes over 18 million cases of severe diseases resulting in over a half million annual deaths [10]. *S. pyogenes* infects many different tissues including the skin, throat, muscle and blood [11]. To cause these infections, *S. pyogenes* not only produces various virulence factors, but also regulates the expression of their genes in an exquisite manner. In *S. pyogenes,* research on the regulation of gene expression has focused mainly on protein regulators such as two-component and stand-alone regulators. Recent studies, however, have revealed that sRNAs also play a crucial role in *S. pyogenes* pathogenesis [12], [13], [14], [15], [16].

Compared to the number of *E. coli* sRNAs discovered so far (more than 80 [17]), the number of experimentally verified *S. pyogenes* sRNAs is relatively small. Only 17 sRNAs have been validated with mostly Northern blotting [12], [13], [14], [16], [18], [19], [20] or mutational analysis [15]. This implies that systematic genome-wide search for sRNAs in *S. pyogenes* may not have been sufficiently carried out. In this study, through employing a combination of three computational algorithms and Northern blotting, we discovered 7 novel sRNAs in *S. pyogenes*.

## Methods

### Bacterial strains and media

*S. pyogenes* MGAS315 [21] was used for most experiments. MGAS315 is a non-mucoid clinical strain isolated from a patient with streptococcal toxic shock syndrome, and its genome sequence is publically available [21]. *S. pyogenes* was routinely cultured in Todd-Hewitt medium (BBL) supplemented with 0.2% yeast extract (Difco) at 37°C in sealed tubes without agitation.

### Computational analysis for the screening of putative *S. pyogenes* small RNAs

**Figure 1:**
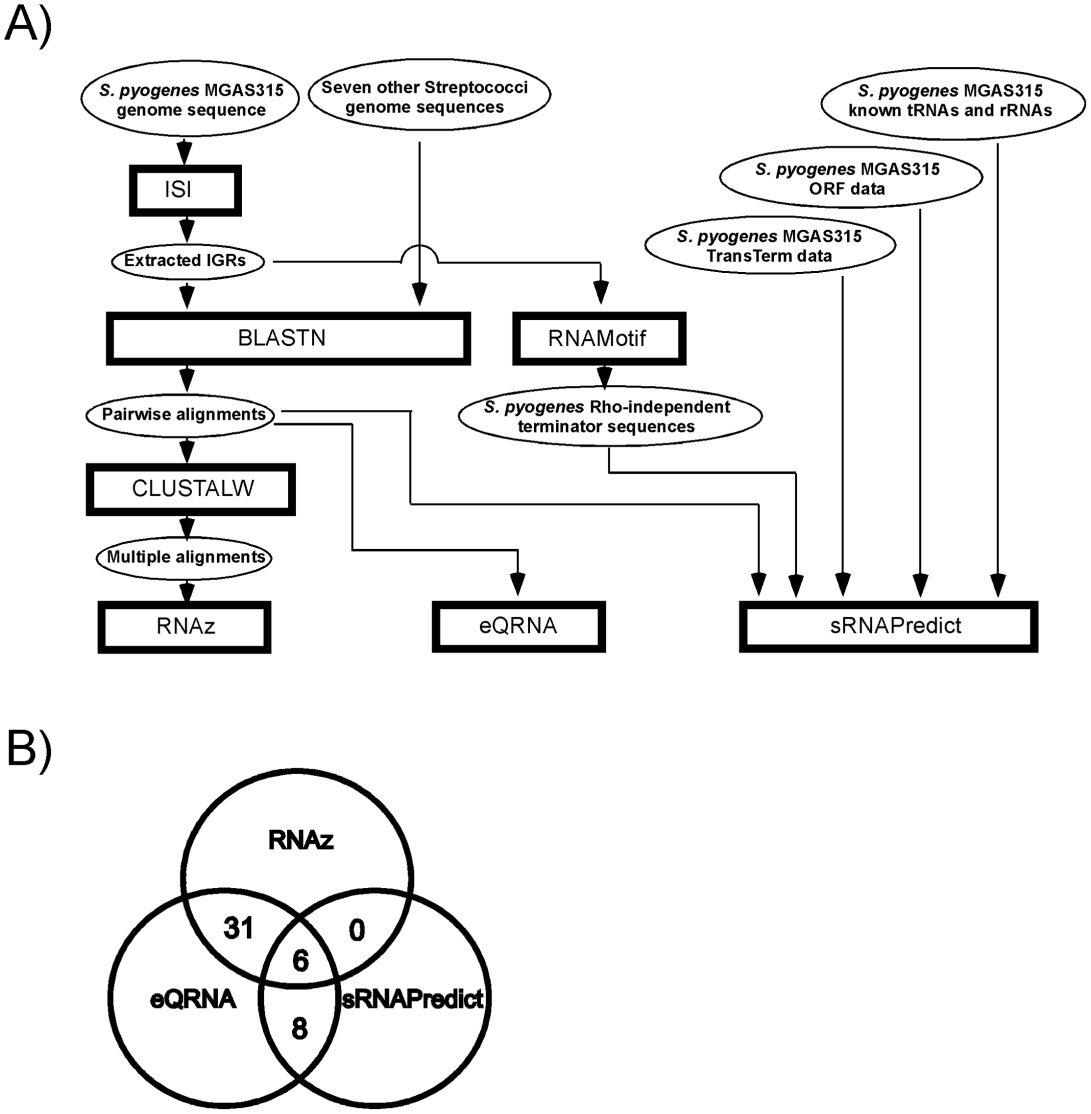
A combination of three computational algorithms was used to predict small regulatory RNAs in *S. pyogenes*. A) The scheme of the computational approach for the prediction of small RNAs in *S. pyogenes*. The rectangles, ovals, and arrow lines represent computational algorithms, input or output data of computational analyses, and data flow, respectively. The processes were performed to run the algorithms, RNAz, eQRNA and sRNAPredict. B) The candidates predicted by any two algorithms at the same time were considered sRNA candidates. Then, putative *cis*-regulatory sequences located immediately upstream of annotated ORFs and candidates within prophage sequences were removed from the candidate list. The number of final candidates selected in this manner was 45.

In this study, we employed three computational algorithms to increase prediction accuracy: eQRNA, RNAz, and sRNAPredict. The computational approaches are illustrated in Figure 1 and the sources and references of the computational algorithms used in this study are listed in Table 1. The genomic information of the following eight streptococci necessary to run these algorithms, such as genome sequences, loci and names of open reading frames (ORFs), tRNAs, tmRNA, rRNAs and IGRs, was downloaded from the NCBI website (ftp://ftp.ncbi.nih.gov/genomes/Bacteria/): *S. pyogenes* MGAS315, *S. equi subsp. zooepidemicus* MGCS10565, *S. mutans* UA159, *S. suis* 05ZYH33, *S. sanguinis* SK36, *S*. *gordonii str. Challis substr.* CH1, *S. pneumoniae* CGSP14, *S. agalactiae* NEM316.

**Table 1:** The list of computational algorithms used in this study.

Computational Method used	Source	Ref
ISI	http://www.biochpharma.univ-rennes1.fr/	[61]
WU-BLAST (BLAST 2.0)	http://blast.wustl.edu	[62]
RNAz 1.0	http://www.tbi.univie.ac.at/~wash/RNAz/	[35]
ClustalW 2.0.11	http://www.ebi.ac.uk/Tools/clustalw2/index.html	[63]
eQRNA 2.0.3c	ftp://selab.janelia.org/pub/software/qrna/	[34]
RNAMotif 3.0.5	http://casegroup.rutgers.edu/	[64]
sRNAPredict 3	http://newbio.cs.wisc.edu/sRNA/	[29], [30]
TransTerm 2.07	http://transterm.cbcb.umd.edu	[65]

To run sRNAPredict, the coordinates and the orientations of 1865 ORFs, 67 tRNAs, 18 rRNAs and 1tmRNA in the genome of *S. pyogenes* MGAS315 were marked and then 1587 IGRs were extracted by Intergenic Sequence Inspector (ISI). To predict the location of Rho-independent terminators of *S. pyogenes* MGAS315, the RNAmotif program and the TransTerm database were used. RNAMotif searches RNA sequences that match a “motif” describing the interactions of secondary structures, which are defined via a pattern language whose symbols represent helices and single stranded stretches. Matches can be ranked by applying scoring rules that may provide finer distinctions than just matching to a profile. Rho-independent terminators predicted by TransTerm were obtained from the TransTerm website at University of Maryland. Putative terminators whose probabilities (confidence) were greater than 90% were chosen for sRNAPredict analysis. Parameters applied to sRNAPredict analysis are as follows; the minimum distance of predicted terminator from the end of an upstream ORF: 30 nts, the maximum length of gap between a putative terminator and a region of sequence conservation: 20 nts, the values for the minimum and maximum length of putative sRNAs: 30 nts and 550 nts.

For the prediction with eQRNA and RNAz, a file containing intergenic regions (IGRs) of *S. pyogenes* MGAS315 was generated using ISI. The number of the extracted IGRs from *S. pyogenes* MGAS315 genome by ISI was 3166 sequences from both DNA strands, totaling about 0.681 MB which represents 17.9% of the full genome. The average sequence length was 138 nts, with the longest one being 1501 nts and the shortest one being 1 nt. Then, the WU-BLAST 2.0 program was used for genome wide sequence homology analysis between IGRs of *S. pyogenes* MGAS315 and the genomes of the other seven streptococci. The IGRs with a length >12 nts were used as queries. The output data of the BLAST analysis gave pairwise alignments between the query sequences, that are the IGRs of *S. pyogenes* MGAS315, and the subject sequences, that are the genomic segments of the other seven streptococci. These pairwise alignments of 2118 comparisons were filtered with the parameters of *E*-values <0.00001 and length >30 nts (Table S1). These pairwise alignments were scanned by CLUSTALW to produce multiple alignments. From the multiple alignments, the long sequences with the length >550 nts were removed. Alignments contained sequences from both DNA strands, and candidates were selected when a signal was identified from either of the two strands. RNAz and eQRNA predictions from the alignments were incorporated into a single predicted RNA locus on the genome. An additional set of alignments was obtained using 69 known RNAs (68 tRNAs and one tmRNA) as queries. These RNA alignments were generated using the same BLAST parameters, and were used to evaluate the sensitivity and specificity of those computational analyses. The multiple sequence alignments that were formatted to CLUSTALW data were used as the input source for RNAz. The CLUSTALW program uses FASTA format as input data. Thus, we transformed the pairwise sequences from BLASTN to FASTA format to execute CLUSTALW. Both RNAz and eQRNA used the window size of 150 nts and the window slide increment of 50 nts. To test sensitivity and specificity of this approach, we used 68 known tRNAs and tmRNA as controls. Through the RNAz and eQRNA analyses, 65 out of 68 *S. pyogenes* tRNAs and tmRNA were identified (95.6% sensitivity) (Table S2). To test the specificity of the RNAz and eQRNA analyses, we shuffled the sequences of the tRNAs and tmRNA and estimated the false positives if any shuffled sequence was considered as sRNA. For shuffling of sequences, the RNAs were divided into several groups and the sequence locations of the groups (each group has 20 nts and the size of an ordinary tRNA is about 70 nts) were exchanged. This shuffling keeps the sequence conservation but not the conservation of secondary structures. The number of false positives obtained from this process was 1/68 (1.47%), so the specificity was 98.5% (Table S2). RNAz analysis shows similar sensitivity but higher specificity than the eQRNA analysis, and the combination of RNAz and eQRNA analyses increased specificity compared to the individual analysis, as expected (Table S2).

The computer algorithms used in this study and the result of each process can be downloaded from www.cs.siu.edu/~nyu/research.htm.

### RNA extraction from *S. pyogenes* MGAS315

Total *S. pyogenes* RNA was extracted using the combination of the miRNeasy kit (Qiagen) and the FastPrep beadbeater (MP biomedicals). An *S. pyogenes* cell pellet from 10 ml culture was resuspended in 700 μl of the Qiazol lysis reagent provided in the miRNeasy kit and transferred to a Lyse Matrix B blue cap tube (MP biomedicals). Cells were then lysed by the beadbeater, FastPrep 24 (MP biomedicals) at the speed of 6.0 for 40 seconds twice. The remaining procedure for RNA extraction followed the manufacturer's protocol of the miRNeasy kit. The A260/A280 ratio of the extracted RNA was measured with NanoDrop (Thermo Scientific) to determine the RNA concentration and purity (accepted if >1.8). The extracted RNA was mixed with 1 μl of RNasin (Promega Recombinant RNasin Ribonuclease Inhibitor, 40 u/μl), and treated with RNase-Free DNase (Promega DNase I, 1u/µl) according to the manufacturer's protocol.

### Northern blot analysis

*S. pyogenes* MGAS315 was grown in THY medium at 37°C and harvested with centrifugation (at 6,000 × g for 3 min) at the exponential phase (OD_600_, Optical density at 600 nm,  = ∼0.5), the early stationary phase (OD_600_  = ∼1.2) or the late stationary phase (cells were grown for 3 hrs more from the early stationary phase, OD_600_  = ∼1.5). Then, Total RNA was extracted as described above. The extracted RNA (20 µg) was mixed with loading dye containing 50% (v/v) formaldehyde, loaded onto denaturing polyacrylamide gel (6% polyacrylamide (acrylamide: bis-acrylamide  = 29∶1) with 7% urea) pre-run at 400 V for an hour, and electrophoresed at 300 V. For an RNA size marker, 5 ng of Low Range SSRCNA Ladder (New England BioLab, 500 μg/ml) was loaded in a well. The separated RNA was transferred onto a nylon membrane (Zeta Probe Blotting Membrane, Bio-Rad) with a semi-dry electroblotter (Bio-Rad Trans-Blot SD Transfer Cell) at 400 mA for two hours at 4°C. The RNA on the nylon membrane was cross-linked to the membrane with 1-ethyl-3-(3-dimethylaminopropyl)-carbodiimide (EDC) [22]. The membrane was then prehybridized with 5X SSPE/2% SDS hybridization buffer for 30 min and hybridized for 18 hrs with the same buffer containing a 40 nM single stranded DNA oligonucleotide probe (Table S3). The probe had been ^32^P-labeled at 5′-end with γ-^32^P ATP (10 mCi/ml, PerkinElmer) by T4 polynucleotide kinase (Epicentre Technologies). The probes were designed to bind at the center of putative small RNAs predicted by the computational algorithms. Because the list of sRNA candidates from the computational analysis did not provide information on which DNA strand encodes each sRNA candidate, we designed and tested two probes annealing to each strand. The sequence of each probe is shown in Table S3. After hybridization, the membrane was washed with wash solutions and exposed to X-ray film (autoradiography).

### Reverse transcriptase PCR (RT-PCR)

*S. pyogenes* MGAS315 was grown in THY medium at 37°C until it reached an OD_600_ of 0.5. The cells were harvested with centrifugation and total RNA was extracted as described in “RNA Extraction from *S. pyogenes* MGAS315”. The extracted RNA was converted to cDNA using reverse transcriptase (ImProm II Reverse Transcriptase, Promega) according to the manufacturer's protocol. Briefly, RNA (2 µg) was mixed with 500 ng of random primers (Promega) and adjusted to 5 μl with RNase free water. The RNA mixture was incubated at 65°C for five minutes and then chilled at 4°C for five minutes. A mix containing 2.4 μl of MgCl_2_ (25 nM), 4 μl of ImProm-II 5× reaction buffer, 1 μl of ImProm-II Reverse Transcriptase, 1 μl of dNTPs (10 nM) and 6.6 μl of RNase Free water was added to the RNA mix and incubated at 25°C for five minutes, followed by 42°C for one hour, and then heat inactivated at 70°C for fifteen minutes. Regular PCR was performed with the cDNA as a template. RNA (without reverse transcriptase reaction) was used as a control to confirm that a PCR product was not from chromosomal DNA contamination (RNA was rejected before producing cDNA if PCR amplification performed with the RNA template indicated the presence of contaminating DNA). PCR products and a DNA ladder (1 Kb Plus DNA Ladders, Invitrogen) were electrophoresed on a 2% agarose gel.

Primers for RT-PCR were designed with the parameters of 1 bp GC clamp at 3′ end, 20 nts size, 100–200 nts product size, and 60°C melting temperature (Table S4).

### Circular RACE to determine sRNA transcriptional start and stop sites

The transcriptional start and stop sites of sRNA candidates were determined using circular RACE (Rapid Amplification of cDNA ends) as described elsewhere [23]. Briefly, RNA was extracted from *S. pyogenes* cultures in the exponential phase (OD_600_ of 0.5) as described above. The RNA was treated with Tobacco Acid Pyrophosphatase (TAP) (Epicentre) to remove pyrophosphate from the 5′ end. The 5′ end was then ligated to the 3′ end with T4 RNA ligase (Epicentre) to make circular transcripts. The circular transcripts were reverse transcribed using gene specific primers to make first strand cDNA. The first strand cDNAs were amplified with PCR. The PCR products were cloned into pGEM-T (Promega) and sequenced to determine transcriptional start and stop sites. Primers for the circular RACE used in this study are listed in Table S5.

### Deletion of SSRC21 in the chromosome

To create a deletion mutant of SSRC21, ΔSSRC21cat, 144 bps of the internal part of SSRC21 was deleted and replaced with a chloramphenicol acetyltransferase gene (*cat*) [24] through a double cross over homologous recombination. To achieve this, first, two DNA fragments flanking SSRC21 on each side were amplified with primers and joined to delete SSRC21. The primers 5outSSRC21far and 3inSSRC21 were used to amplify 1.23 kbps DNA fragment upstream of SSRC21, and 5inSSRC21 and 3outSSRC21*Pst*I were used to amplify 0.69 kbps DNA fragment downstream of SSRC21 (Table 2). These two fragments were digested with *Xma*I, ligated, and PCR amplified with the primers 5outSSRC21 and 3outSSRC21*Pst*I (Table 2). The PCR-amplified 1.36 kbps DNA was sequenced to confirm the deletion of SSRC21 and inserted into vector pJRS233 [25]. The deleted SSRC21 was replaced with the *cat* gene (0.98 kbps) that was PCR-amplified from pABG5 [26] with the primers of 5cat*Xma*I and 3cat*Xma*I (Table 2). The *cat* gene flanked with ∼0.7 kbp streptococcal DNAs on each side was amplified with the same primers used to amplify the 1.36 kbp PCR fragment (5outSSRC21 and 3outSSRC21PstI), and then used to transform MGAS315 (wild type) by electroporation. The mutant showing chloramphenicol resistance was selected and the chromosomal structure was confirmed by PCR.

**Table 2:** Primers to be used to create the SSRC21 deletion mutant, ?SSRC21cat.

Name	Sequence#
5outSSRC21far	GGTATTAAAGGATAGCACATCAAC
3inSSRC21	TTT**CCCGGG**CAATCGACTCATCGCATACAG
5inSSRC21	TTT**CCCGGG**ATCTTAGTTAAAATTCAGAATGTATCAG
3outSSRC21PstI	TTT**CTGCAG**GGAGGGGAGTTTCCAAAATG
5outSSRC21	TTT**GGATCC**ATGTGGTCTATCACAGAAAAAGAAC
3outSSRC21PstI	TTT**CTGCAG**GGAGGGGAGTTTCCAAAATG
5catXmaI	AAA**CCCGGG**GGATTTTTCGCTACGCTCAAATC
3catXmaI	AAA**CCCGGG**CTTCTTCAACTAACGGGGCAG

# The restriction enzyme sites in primers are indicated in bold.

### Next-generation sequencing, RNA-Seq

Total RNA was extracted from *S. pyogenes* cultures in the exponential phase (OD_600_ of 0.5) as described above, and submitted to Otogenetics Corporation (Norcross, GA USA) for RNA-Seq assays. Briefly, the integrity and purity of total RNA were assessed using Agilent Bioanalyzer and OD260/280. Up to 5 μg of total RNA was subjected to rRNA depletion using the RiboZero Meta-Bacteria kit (Epicentre Biotechnologies, Madison, WI USA, catalog # MRZMB126) and cDNA was generated from the depleted RNA using the NEBNext mRNA Sample Prep kit (New England Biolabs, Ipswich, MA USA, catalog# E6110). cDNA was profiled using Agilent Bioanalyzer, and subjected to Illumina library preparation using NEBNext reagents (New England Biolabs, Ipswich, MA USA, catalog# E6040). The quality and quantity and the size distribution of the Illumina libraries were determined using an Agilent Bioanalyzer 2100. The libraries were then submitted for Illumina HiSeq2000 sequencing according to the standard operation. Paired-end 90 or 100 nucleotide (nt) reads were generated and subjected to data analysis using the platform provided by Center for Biotechnology and Computational Biology (University of Maryland, College Park, MD USA) as previously described [27]. The data sets generated from RNA-Seq were mapped against GenBank AE014074 (http://www.ncbi.nlm.nih.gov/nuccore/21905618) with Bowtie2 (V2.0.0.5). Hits on regions defined by GenBank were then counted with bedtools. To determine the difference of gene expression between samples, EdgeR (Empirical analysis of digital gene expression data in R) was used. We deposited our RNA seq dataset to NIH Short Read Archive with the accession number SRP020234.

## Results

### Computational analysis to predict small RNAs in *S. pyogenes* MGAS315

Along with experimental strategies based on shotgun cloning or microarray methods, computational predictions and validation with Northern blot have been a popular method used to identify many sRNAs (for a review, refer to [28]). Most computational algorithms developed for genome-wide screening of small RNAs are based on ‘sequence or structural conservation’ among closely related species. The algorithms that seek sequence conservation such as sRNAPredict [29], [30], and GMMI [31] search first for transcriptional signals such as promoters and terminators, and then examine nucleotide conservation. Since the sequence homology information is based only on the primary structure of RNA, the accuracy of this method may not be sufficiently adequate. Hence, some algorithms seek for phylogenetic conservation of secondary structure and/or thermal stability. This type of algorithm includes Pfold [32], MSARI [33], eQRNA [34], RNAz [35], etc. In this study, we employed a combination of three algorithms that search for different forms of conservation as a way to increase prediction accuracy: sRNAPredict [29], [30], eQRNA [34], and RNAz [35] (Figure 1). The sRNAPredict algorithm uses the information of the location of transcriptional signals and primary sequence conservation of intergenic regions (IGRs). The eQRNA algorithm examines the conservation of secondary structures of RNA for prediction. It identifies base substitution patterns in pairwise alignments likely corresponding to a conserved RNA secondary structure rather than to a conserved coding frame or other genomic features. RNAz even measures thermodynamic stability, which is normalized with respect to both sequence length and base composition in addition to RNA consensus secondary structure. The eQRNA and RNAz algorithms are comparatively strict methods for secondary structure conservation analysis compared to other methods such as Pfold [32] and MSARI [33]. To search for those conservations between closely-related streptococcal species, genome sequences and annotations of the following eight streptococci were used. *S. pyogenes* MGAS315, *S. equi subsp. zooepidemicus* MGCS10565, *S. mutans* UA159, *S. suis* 05ZYH33, *S. sanguinis* SK36, *S*. *gordonii str. Challis substr.* CH1, *S. pneumoniae* CGSP14, *S. agalactiae* NEM316.

Each algorithm of sRNAPredict, eQRNA, and RNAz respectively predicted 191, 312, and 187 intergenic genomic segments as putative sRNAs in *S. pyogenes* MGAS315. Among these predicted ones, the sequences immediately upstream of annotated open reading frames (ORFs) were removed from the candidates because they most likely correspond to putative riboswitches or other *cis*-regulatory elements. In addition, predicted candidates in prophages, all of which were located next to the integrase genes, were removed. Then, the intergenic genomic segments predicted by any two algorithms were considered as putative sRNA candidates. The final number of sRNA candidates left from these processes was 45 (Figure 1B, Table 3, and Table S6). Encouragingly, Pel, FasX, and RivX, which have been previously studied sRNAs, were included in these 45 streptococcal small RNA candidates (SSRCs). Among the 45 putative candidates, six (SSRC 8, 12, 15, 30, 32, 33) were predicted by all the three algorithms (Table 3).

**Table 3:** The result of computational screening for sRNA candidates in *S. pyogenes*.

SSRC^a^	Up ORF number	Up ORF name	up ORF^b^	Down ORF number	Down ORF name	down ORF^b^	Method#	Remark and reference
SSRC 1	SPyM3_0029	hypothetical protein	>>>	SPyM3_0030	adenylosuccinate lyase	>>>	Q, Z	
SSRC 2	SPyM3_0054	30S ribosomal protein S8	>>>	SPyM3_0055	50S ribosomal protein L6	>>>	Q, Z	
SSRC 3	SPyM3_0156	glucose-6-phosphate isomerase	>>>	SPyM3_0157	putative regulatory protein RofA related	<<<	Q, Z	RivX [15]
SSRC 4	SPyM3_0174	putative response regulator	>>>	SPyM3_0175	ribonuclease P	>>>	Q, Z	FasX [12]
SSRC 5	SPyM3_0199	30S ribosomal protein S7	>>>	SPyM3_0200	elongation factor G	>>>	Q, Z	
SSRC 6	SPyM3_0201	glyceraldehyde-3-phosphate dehydrogenase	>>>	SPyM3_0202	putative amino acid ABC transporter ATP-binding protein	<<<	Q, Z	
SSRC 7	SPyM3_0219	putative oligopeptide ABC transporter	>>>	rRNA	rRNA	>>>	Q, P	
SSRC 8*	SPyM3_0298	putative cell envelope proteinase	>>>	SPyM3_0299	hypothetical protein	>>>	Q, Z, P	
SSRC 9	SPyM3_0300	methionyl-tRNA synthetase	>>>	SPyM3_0301	ribonucleotide-diphosphate reductase subunit beta	>>>	Q, Z	
SSRC10	SPyM3_0439	hypothetical protein	>>>	SPyM3_0440	putative calcium transporter	>>>	Q, P	
SSRC11	SPyM3_0455	putative cell-division protein	>>>	SPyM3_0457	putative metallo-beta-lactamase superfamily protein	<<<	Q, Z	
SSRC12*	SPyM3_0480	streptolysin S associated protein	>>>	SPyM3_0481	streptolysin S associated protein	>>>	Q, Z, P	Pel [13], [14]
SSRC13	SPyM3_0505	putative DNA-entry nuclease	>>>	SPyM3_0506	phenylalanyl-tRNA synthetase subunit alpha	>>>	Q, Z	
SSRC14	SPyM3_0557	putative ribosomal large subunit pseudouridine synthase	>>>	SPyM3_0558	bifunctional pyrimidine regulatory protein	>>>	Q, P	
SSRC15*	SPyM3_0582	putative peptidoglycan hydrolase	>>>	SPyM3_0583	hypothetical protein	<<<	Q, Z, P	
SSRC16	SPyM3_0588	putative ABC transporter ATP-binding protein	>>>	SPyM3_0589	hypothetical protein	>>>	Q, Z	
SSRC17	SPyM3_0611	purine nucleoside phosphorylase	>>>	SPyM3_0612	putative purine nucleoside phosphorylase	>>>	Q, Z	
SSRC18	SPyM3_0663	branched-chain alpha-keto acid dehydrogenase subunit E2	>>>	SPyM3_0664	putative dihydrolipoamide dehydrogenase component E3	>>>	Q, Z	
SSRC19	SPyM3_0664	putative dihydrolipoamide dehydrogenase component E3	>>>	SPyM3_0665	extracellular hyaluronate lyase	<<<	Q, Z	
SSRC20	SPyM3_0762	2-amino-4-hydroxy-6-hydroxymethyldihydropteridine pyrophosphokinase	>>>	SPyM3_0763	UDP-N-acetylenolpyruvoylglucosamine reductase	>>>	Q, P	
SSRC21	SPyM3_0851	putative anaerobic ribonucleotide reductase	<<<	SPyM3_0852	putative cardiolipin synthetase	<<<	Q, P	[20], [42]
SSRC22	SPyM3_0918	hypothetical protein	<<<	SPyM3_0919	hypothetical protein	<<<	Q, Z	
SSRC23	SPyM3_0983	putative maltose/maltodextrin-binding protein	>>>	SPyM3_0984	putative maltose/maltodextrin ABC transport system (permease)	>>>	Q, Z	
SSRC24	SPyM3_0989	hypothetical protein	<<<	SPyM3_0990	putative esterase	<<<	Q, Z	
SSRC25	SPyM3_1093	putative heavy metal/cadmium-transporting ATPase	<<<	SPyM3_1094	hypothetical protein	<<<	Q, Z	
SSRC26	SPyM3_1166	isoleucyl-tRNA synthetase	<<<	SPyM3_1167	putative cell-division initiation protein	<<<	Q, Z	
SSRC27	SPyM3_1176	UDP-N-acetylmuramoyl-L-alanyl-D-glutamate synthetase	<<<	SPyM3_1177	hypothetical protein	<<<	Q, P	
SSRC28	SPyM3_1190	asparagine synthetase AsnA	<<<	SPyM3_1191	carbamate kinase	<<<	Q, Z	
SSRC29	SPyM3_1276	hypothetical protein	<<<	SPyM3_1277	hypothetical protein	<<<	Q, P	[20], [42]
SSRC30*	SPyM3_1280	3-dehydroquinate synthase	>>>	SPyM3_1281	putative acetate kinase	<<<	Q, Z, P	
SSRC31	SPyM3_1356	hypothetical protein	>>>	SPyM3_1357	hypothetical protein	>>>	Q, Z	
SSRC32*	SPyM3_1386	putative N6-adenine-specific DNA methylase	<<<	SPyM3_1387	hypothetical protein	<<<	Q, Z, P	[20], [42]
SSRC33*	SPyM3_1391	putative aminopeptidase C	<<<	SPyM3_1392	NAD synthetase	<<<	Q, Z, P	
SSRC34	SPyM3_1510	hypothetical protein	>>>	SPyM3_1511	putative mannose-specific phosphotransferase system component IIAB	>>>	Q, Z	
SSRC35	SPyM3_1533	heat-inducible transcription repressor	<<<	SPyM3_1534	N-acetylmuramoyl-L-alanine amidase	<<<	Q, Z	
SSRC36	SPyM3_1644	putative deoxyribonuclease hsdM modification subunit	>>>	SPyM3_1645	putative response regulator of salavaricin regulon	<<<	Q, Z	
SSRC37	SPyM3_1673	hypothetical protein	<<<	SPyM3_1674	putative serine acetyltransferase	<<<	Q, Z	
SSRC38	SPyM3_1725	laminin-binding protein	<<<	SPyM3_1726	C5A peptidase precursor	<<<	Q, Z	
SSRC39	SPyM3_1726	C5A peptidase precursor	<<<	SPyM3_1727	antiphagocytic M protein, type 3	<<<	Q, Z	[40]
SSRC40	SPyM3_1766	co-chaperonin GroES	<<<	SPyM3_1767	putative endopeptidase Clp ATP-binding chain C	<<<	Q, P	
SSRC41	SPyM3_1798	hypothetical protein	<<<	SPyM3_1799	transcriptional regulator Spx	<<<	Q, Z	
SSRC42	SPyM3_1817	50S ribosomal protein L33	>>>	SPyM3_1818	putative cadmium resistance protein	>>>	Q, Z	[40]
SSRC43	SPyM3_1822	hypothetical protein	>>>	SPyM3_1823	hypothetical protein	<<<	Q, Z	
SSRC44	SPyM3_1836	hypothetical protein	<<<	SPyM3_1838	tRNA uridine 5-carboxymethylaminomethyl modification enzyme GidA	<<<	Q, Z	
SSRC45	SPyM3_1843	hypothetical protein	<<<	SPyM3_1844	putative ABC transporter membrane-spanning permease	<<<	Q, Z	

a SSRC: *S. pyogenes* Small RNA Candidate. The nucleotide coordinates of SSRC predicted by each computational algorithm are listed in Table S5.

b Genes present on the strand given in the *S. pyogenes* MGAS315 genome databases are indicated by >>> and those on the complementary strand by <<<.

# Algorithms that identify each sRNA candidate: Q, eQRNA; Z, RNAz; P, sRNAPredict.

* sRNA candidates predicted by all the three algorithms, eQRNA, RNAz, and sRNAPredict.

### Verification of the transcription of the predicted sRNA candidates through reverse transcriptase PCR (RT-PCR) and Northern blotting

Before applying Northern blotting to verify the expression of streptococcal sRNA candidates, we screened the candidates with RT-PCR. RT-PCR can at least detect the presence or absence of their transcripts, even though it cannot distinguish whether the expressed transcripts are *cis*-elements attached to mRNAs or independently expressed sRNAs. In the RT-PCR analysis, we did not detect the expression of SSRC 1, 3, 6, 7, 22, 24, 28, 39, 40, so we eliminated them from the sRNA candidates.

Then, we examined the remaining candidates with Northern blotting. Since most *S. pyogenes* sRNAs previously described were highly expressed in the exponential phase of growth [20], RNA was extracted from cells at the exponential phase and used for Northern blotting. The predicted sRNA candidates could be expressed from either strand of DNA, so we designed and tested two probes for each candidate (Table S3).

**Figure 2:**
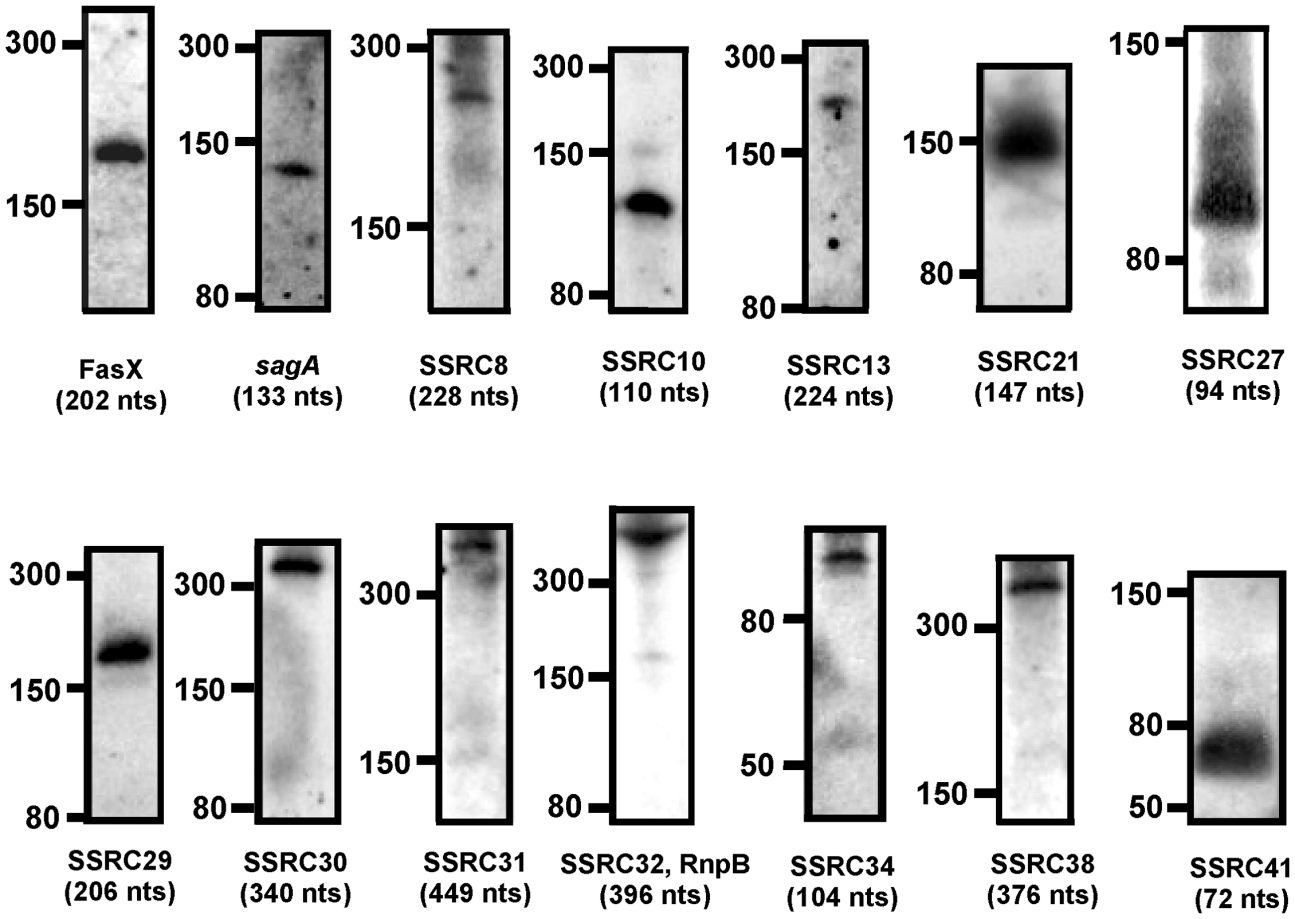
Northern blot identified *S. pyogenes* sRNAs from the candidates predicted by the computational analysis. Northern blots were performed with RNA (20 µg) extracted from MGAS315 at the exponential growth phase (Optical density at 600 nm, OD_600_, ∼0.5). The names of the candidate RNA molecules are shown at the bottom of each Northern blot as SSRC (Streptococcal Small RNA Candidate) number. The locations of size markers in nucleotides are shown at the left side of each Northern blot. The approximate sizes of SSRCs calculated based on the location of the size markers are shown in nucleotides (nts) below their names.

Among the candidates, the following 14 SSRCs showed consistent signals on Northern blots: SSRC4 (FasX), SSRC8, SSRC10, SSRC12 (Pel/*sagA*), SSRC13, SSRC21, SSRC27, SSRC29, SSRC30, SSRC31, SSRC32, SSRC34, SSRC38, and SSRC41 (Figure 2). Some candidates (detected with 5′ probes in the Table S3) were expressed from the top DNA strand of the MGAS315 chromosome sequence (GenBank: AE014074.1, http://www.ncbi.nlm.nih.gov/nuccore/21905618?report=fasta) and the others (detected with 3′ probes in the Table S3) were from the complementary DNA strand; The candidates, SSRC21, SSRC27, SSRC29, SSRC30, SSRC32, and SSRC34 were detected with their 5′ Northern blot probes, and SSRC8, SSRC10, SSRC13, SSRC31, SSRC38, and SSRC41 were detected with their 3′ probes. FasX and Pel/*sagA* were detected as expected. However, RivX was not detected probably because of its extremely low expression in the wild type [15]. Since FasX gave a constant signal, it was used as a control throughout the Northern blot analysis.

The size of each SSRC detected by Northern blot was calculated by measuring traveled distance of each band from a well of a polyacrylamide gel and compared to a standard curve obtained from the RNA ladder that was run with each sRNA side by side. The calculated approximate sizes of the SSRCs are shown in Figure 2. The calculated size of FasX (202 nts) was very close to the actual size (203 nts). The probe designed for Pel/*sagA*-transcript detected a 133 nts band only, which is the size close to the *sagA*-transcript, not Pel (459 nts).

### Sequence analysis of the small RNAs detected by Northern blotting

Sequence analyses based on the predicted SSRC sequences (Table S6) and next-generation sequencing, RNA-Seq, revealed that SSRC13, SSRC31 and SSRC32 are probably not *trans*-acting regulatory sRNAs. SSRC13 and SSRC31 appear to be *cis*-elements in RNA-Seq analysis. The sequence of SSRC13 contains a T-box leader element, which is typically found upstream of aminoacyl-tRNA synthetase genes and some amino acid biosynthesis genes and involved in the regulation of those genes' expression by forming a transcription anti-terminator when uncharged tRNA binds the leader sequence [36]. SSRC13 is located upstream of phenylalanyl-tRNA synthetase subunit alpha. SSRC32 appears to be ribonuclease P (RNase P) RNA, RnpB. RNase P is a ribozyme, which is composed of two components, RnpA (protein) and RnpB (RNA), and cleaves the 5′ leader sequence of precursor tRNAs to produce mature tRNAs [37].

**Figure 3:**
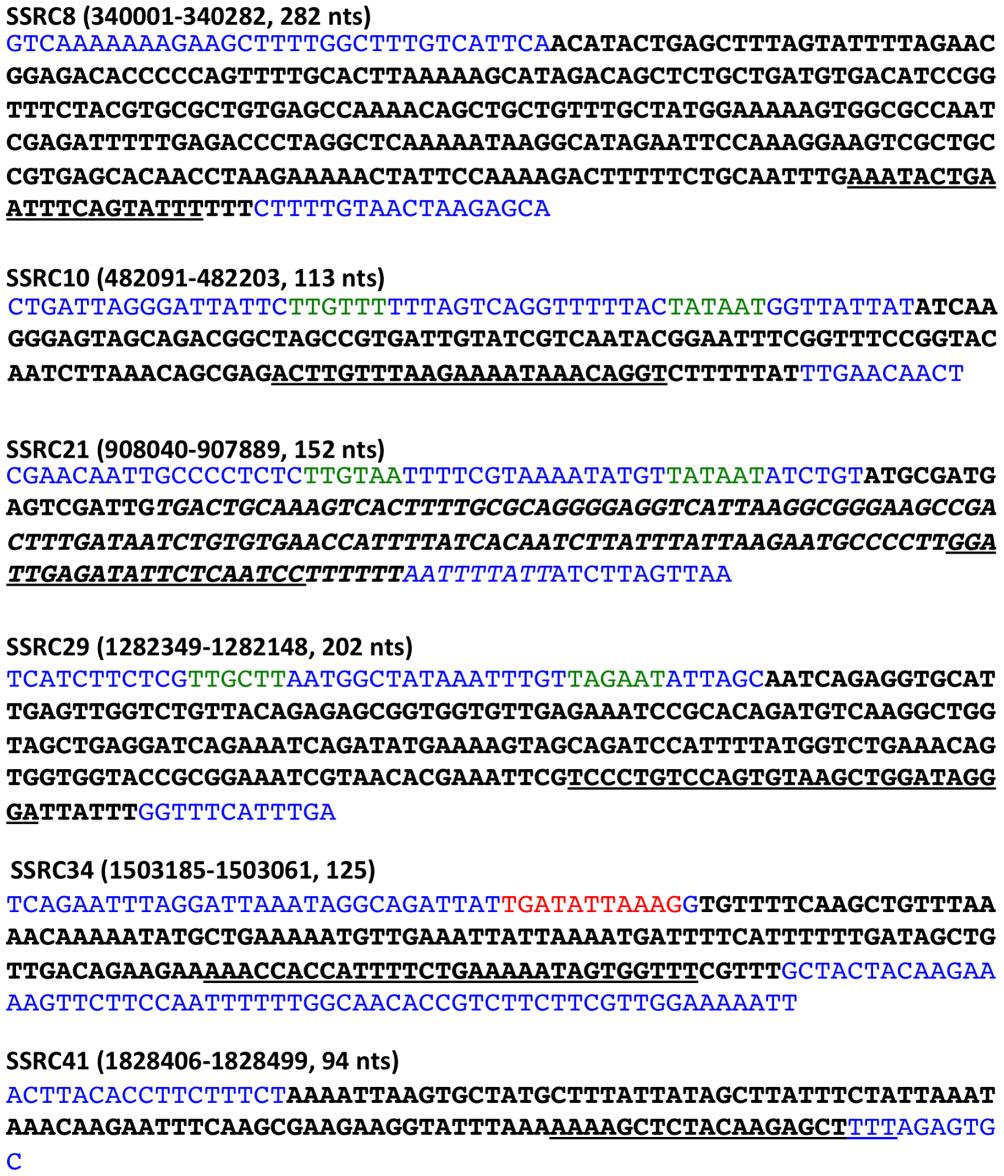
Sequence analysis of candidate sRNA transcriptional start and stop sites, promoter regions and terminators. The transcriptional start and stop sites of candidate sRNAs were determined by circular RACE. The sRNA sequences based on the transcriptional start and stop sites are in black. The putative −10 and −35 promoter sequences are colored green, and putative Rho-independent terminators, which are identified by the algorithm ARNold (http://rna.igmors.u-psud.fr/toolbox/arnold/index.php), are underlined. Neighboring sequences of the sRNA sequences are colored in blue. The deleted part in the SSRC21 deletion mutant, ΔSSRC21cat, is italicized. A putative CovR-binding site upstream of SSRC34 is colored in red. The nucleotide coordinates based on the genome sequence of *S. pyogenes* MGAS315 and sizes of the sRNAs are shown in parenthesis.

We performed circular RACE (Rapid Amplification of cDNA Ends) to determine the 5′ and 3′ ends of selected small RNA candidates. Based on the calculated sizes, Northern blot probe-binding sites, and/or putative transcriptional signatures (promoters and Rho-independent terminators), we designed primers for each SSRC. From this analysis, we could determine the sequences of SSRC8, SSRC10, SSRC21, SSRC29, SSRC34 and SSRC41 (Figure 3). All the determined sequences contained a Rho-independent transcriptional termination signal (hairpin structure ending with or followed by thymidines). By examining the sequence of promoter regions, we could map putative −10 and −35 promoter sequences of SSRC10, SSRC21, SSRC29, and a putative CovR-binding sequence [38] upstream of SSRC34 (Figure 3).

The three computer algorithms used in this study compared sequences between closely related streptococcal species to search for putative sRNAs, so homologs of the newly discovered small RNA would exist in not only *S. pyogenes* but also other streptococcal pathogens. Thus, we searched for the homologs of the SSRCs in the other streptococci whose genome sequences are available publically. Each streptococcal genome was blasted against the SSRC sequences determined through circular RACE. As expected, homologs of the SSRCs existed in many streptococcal bacteria, and more SSRC homologs were found in streptococci more closly related to *S. pyogenes* (Table 4) [39]. For example, *S. dysgalactiae* subsp. *equisimilis*, *S. equi*, *S. parauberis, S. salivarius*, *S. thermophilus* had more than 4 homologs. Notably, *S. dysgalactiae* subsp. *equisimilis*, which is a beta-hemolytic streptococcus very closely related to *S. pyogenes*, contained all the six SSRCS. Exceptionally, some closely related streptococci such as *S. canis* and *S. iniae* did not contain any homolog of the SSRCs. The most frequent SSRC found in streptococci among the six SSRCs was SSRC10.

**Table 4:** The presence of homologs of SSRCs in other streptococci.

*Streptococcus* spp.	SSRC8	SSRC10	SSRC21	SSRC29	SSRC34	SSRC41
*S. agalactiae* NEM316		73% (1–109)	75% (1–100)	74% (25–176)		
*S.dysgalactiae subsp. equisimilis* GGS_124	77% (149–210)	97% (1–113)	91% (1–152)	98% (1–198)	74% (3–195)	86% (1–94)
*S. equi subsp. equi* 4047		96% (1–112)	79% (1–152)	80% (1–180)	72% (3–125)	79% (5–94)
*S. equi subsp. zooepidemicus* MGCS10565		94% (1–110)	79% (1–152)	79% (3–180)	71% (1–90)	78% (1–90)
*S. gallolyticus* UCN34	81% (154–247)	81% (36–111)		88% (25–81)		
*S. gordonii str. Challis substr*. CH1		70% (2–109)	69% (1–90)			
*S. infantarius subsp. infantarius*	83% (154–227)	74% (2–111)		89% (25–81)		
*S. intermedius* JTH08	76% (151–224)	72% (2–111)		66% (2–149)		
*S. lutetiensis*	83% (154–227)					
*S. macedonicus* ACA-DC 198	78% (142–250)	83% (36–111)		86% (25–81)		
*S. mitis* B6	81% (152–229)	80% (56–111)	74% (2–100)			
*S. mutans* UA159	78% (155–235)	77% (1–109)		69% (3–176)		
*S. oralis* Uo5	76% (151–224)	69% (2–111)	73% (1–100)			
*S. parasanguinis* ATCC 15912	79% (144–210)	80% (2–89)	76% (1–50)			
*S. parauberis* KCTC 11537	76% (152–210)	89% (2–113)	86% (1–105)	84% (1–60)		81% (2–53)
*S. pasteurianus* ATCC 43144	85% (142–228)	89% (2–113)	86% (1–105)	84% (1–60)		81% (2–53)
*S. pneumoniae* CGSP14		67% (2–106)				
*S. pseudopneumoniae* IS7493	76% (151–242)	68% (2–111)	74% (1–110)			
*S. salivarius* JIM8777	76% (154–242)	77% (29–111)	78% (1–50)	80% (3–87)		
*S. sanguinis* SK36		68% (2–109)	70% (2–100)			
*S. suis* BM407		73% (1–111)		74% (3–179)		
*S. thermophilus* CNRZ1066	83% (152–209)	80% (29–111)	78% (1–51)	80% (3–87)		
*S. uberis* 0140J		91% (1–113)	83% (1–103)	69% (1–201)		74% (2–94)

• This analysis was performed using the nucleotide BLAST tool in the NCBI website (http://blast.ncbi.nlm.nih.gov/Blast.cgi). Each genome was blasted against the SSRC sequences (Figure 3).

• The numbers (in %) in the cells indicate the identity between the homologous sequences identified by BLAST. The compared sequence in the BLAST result is indicated in parenthesis as nucleotide numbers in SSRC.

• The blank cells in the table indicate that no homologous sequence of more than 50 nucleotides was found.

• Other streptococci listed below did not show any homologous sequence to SSRCs: *S. anginosus F0211, S. australis* ATCC 700641, *S. caballi* DSM 19004, *S. canis* FSL Z3-227, *S. castoreus* DSM 17536, *S. constellatus* subsp. *pharyngis* SK1060, *S. criceti* HS-6, *S. cristatus* ATCC 51100, *S. devriesei* DSM 19639, *S. didelphis* DSM 15616, *S. downei* F0415, *S. entericus* DSM 14446, *S. ferus* DSM 20646, *S. henryi* DSM 19005, *S. hyovaginalis* DSM 12219, *S. ictaluri* 707–05, *S. infantis*, *S. iniae* 9117, *S. macacae* NCTC 11558, *S. marimammalium* DSM 18627, *S. massiliensis* 4401825, *S. merionis* DSM 19192, *S. minor* DSM 17118, *S. orisratti* DSM 15617, *S. ovis* DSM 16829, *S. peroris* ATCC 700780, *S. plurextorum* DSM 22810, *S. porci* DSM 23759, *S. porcinus* str. Jelinkova 176, *S. pseudoporcinus* SPIN 20026, *S. ratti* DSM 20564, *S. sobrinus*, *S. thoraltensis* DSM 12221, *S. tigurinus* AZ_3a, *S. urinalis* 2285–97, *S. vestibularis* F0396.

### The intracellular abundance of the putative *trans*-acting SSRCs varied between different growth phases

**Figure 4:**
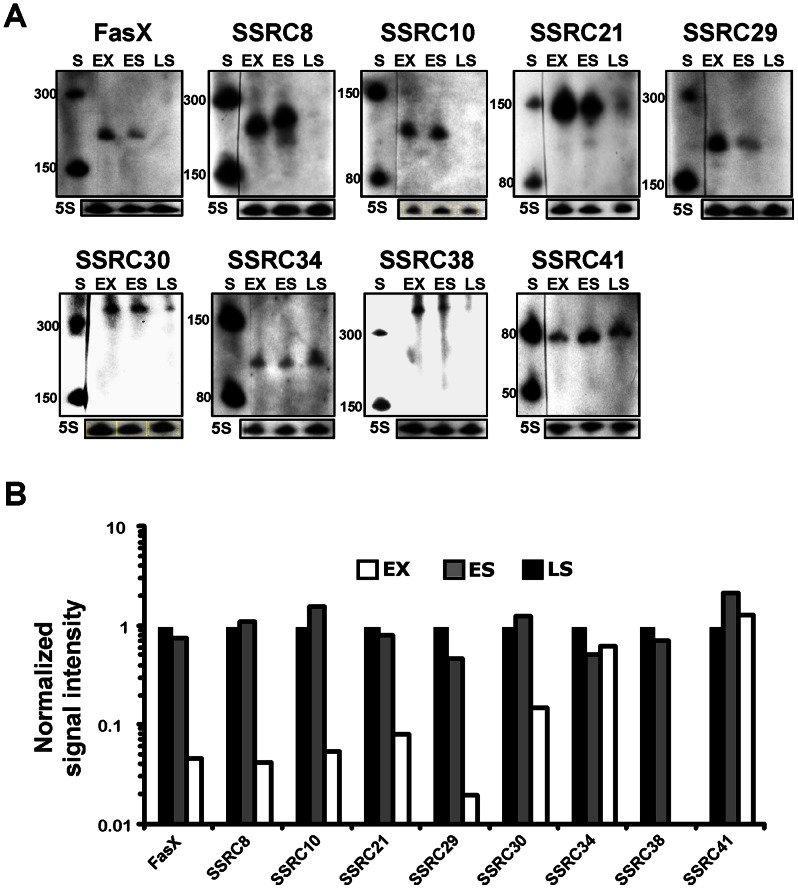
The abundance of newly discovered streptococcal small RNA candidates (SSRCs) varied between growth phases. A) The intracellular abundance of SSRCs at different growth phases. The abundance of each SSRC was determined over the course of growth (exponential phase, EX; early stationary phase, ES; late stationary phase, LS) through Northern blotting. Size markers (S) were run and their sizes are indicated at the left sides of Northern blots. The abundance of 5S RNA (5S) was also determined as a loading control and shown below each Northern blot. B) Abundance of each intracellular sRNA relative to that at the exponential growth phase. Abundance of sRNAs on Northern blots was determined by densitometry, normalized with the abundance of 5S RNA, and expressed relative to the abundance at the exponential growth phase.

We determined the intracellular abundance of the putative *trans*-acting SSRCs at the exponential (EX), early stationary (ES), and late stationary (LS) growth phases of cells through Northern blotting (Figure 4). Agreeing with a previously reported result, FasX was most abundant at the exponential phase [20]. Most SSRCs exhibited variation of abundance between growth phases and were expressed abundantly at the exponential and early stationary phase and least abundantly at the late stationary phase. The abundance of SSRC 8, 10, 21, 29, 30, and 38 was dramatically reduced at the late stationary phase, showing a similar pattern to that of the FasX transcript over the course of growth. On the other hand, SSRC 34 and 41 exhibited similar abundance throughout all growth phases.

### mRNA transcripts showing differential abundance in the SSRC21 deletion mutant

To screen putative target mRNAs of an sRNA detected in this study, we employed differential RNA sequencing analysis. SSRC21 was chosen for this analysis because its expression was changed between growth phases, and initial computational prediction suggested that SSRC21 might influence the expression of several virulence factors. The transcript abundance profile in an SSRC21 deletion mutant was determined through Next-Generation Sequencing, RNA-Seq, and compared to that of the wild type MGAS315. To construct the SSRC21 deletion mutant, ΔSSRC21cat, the SSRC21 gene in the chromosome was replaced with the chloramphenicol acetyl transferase gene, *cat*. In the RNA-Seq result, 142 transcripts exhibited differential abundance with the criteria of fold change in the mutant over the wild type, FC, greater than 2 or less than −2, *p*<0.01, and false discovery rate, FDR, <5% (Table S7). The transcripts (with known putative function) showing notable differential abundance between the mutant and the wild type were those of *ntp* genes encoding V-type ATPase subunits and their putative regulator (SpyM3_0113 through SpyM3_0122; fold changes in the mutant over wild type, FC, 4.2∼7.4). Some transcripts encoding virulence factors also showed differential abundance: streptokinase A, FC 2.6; C5A peptidase, FC −2.1; M protein, FC −2.4; streptococcal phospholipase A2, FC −4.5).

## Discussion

In this study, we discovered new putative *trans*-acting sRNA candidates in the human pathogen *S. pyogenes* using a combination of a bioinformatic prediction and verification with Northern blotting. To overcome the limitation of low accuracy of computational sRNA prediction, we employed three algorithms (sRNAPredict, eQRNA, and RNAz) that seek different kinds of conservations in sRNAs. Since only six were predicted as sRNA candidates by all the three algorithms, we also considered as sRNA candidates those that were predicted by any two algorithms. This resulted in 45 candidates, and the previously studied *S. pyogenes* sRNAs, Pel, FasX and RivX were included among them. Then, we examined their expression through reverse transcriptase PCR and Northern blotting and verified the expression of 14 candidates. In the end, this study added 7 new sRNAs, which are likely trans-acting, to the pool of streptococcal sRNAs, offering a significant source for future study of the role of sRNAs in *S. pyogenes* and related streptococcal pathogens.

Previously, there have been attempts to predict *S. pyogenes* sRNA employing bioinformatics. However, previous studies were not as complete as ours: none of them verified their predictions through Northern blot. Livny *et al*. developed sRNAPredict and predicted sRNA-encoding genes in 10 bacterial pathogens including *S. pyogenes* [29]. However, their prediction missed the previously well-studied *S. pyogenes* sRNAs, Pel, FasX and RivX, indicating low prediction accuracy. There were also other studies using a combination of algorithms to predict *S. pyogenes* small RNAs [40], [41]. However, none of them verified the predicted candidate using Northern blot. Raasch *et al.* [41] used reverse-transcriptase PCR to verify 4 putative candidates. However, RT-PCR cannot distinguish *cis*-acting RNAs such as riboswitches from independently expressed *trans*-acting small RNAs.

Previously, Perez *et al*. performed a genome wide search for *S. pyogenes* sRNA through a microarray-based analysis, and verified14 new sRNAs with Northern blot [20]. Among the 14 sRNAs, only two (SR914400 and SR1251900) overlapped with our putative *trans*-acting sRNA candidates (SSRC21 and SSRC29). Thus, the two methods, microarray and computational prediction followed by Northern blot, appear to identify different sRNAs from each other. A possible explanation for this is that the microarray-based assay might not detect sRNAs with low expression. The expression of sRNAs is generally lower than that of the genes encoding house-keeping proteins, so the signals of sRNAs could be masked by higher signals of house-keeping gene transcripts. Another possibility is the expression difference of sRNAs between strains. The strain used in this study (MGAS315) was different from the strain used by Perez and coworkers (MGAS2221, M1 serotype). Perez and coworkers observed differences of sRNA expression between strains [20]. We also observed the expression difference of an sRNA between strains; the abundance of FasX in HSC5 (M14 serotype) was more than 10 times higher than that of MGAS315 (M3 serotype) at the growth condition used in this study (unpublished data). Recently, Patenge and coworkers published a paper that identified small non-coding RNAs in *S. pyogenes* M49 strain using intergenic tiling array [42]. In their study, only the same two sRNAs identified by Perez *et al.* (SSRC21, SSRC29) overlapped with our sRNAs.

Several sRNAs in *S. pyogenes* have been studied previously. The Pel (pleiotropic effect locus) RNA, which comprises *sagA*, the structural gene of the hemolysin streptolysin S (SLS) [14], has been reported to regulate the expression of Emm (M protein), Sic (streptococcal inhibitor of complement), Spn (*S. pyogenes* NAD(+) glycohydrolase) and SpeB (streptococcal cysteine protease) [13], [14]. However, other studies could not recapitulate the previous studies [18], [20], [43], thus the influence of Pel on the expression of the virulence factors would be strain-specific. In our study, we only detected *sagA* mRNA, not Pel. This is probably because of the use of exponential phase cells in our study. Pel in MGAS315, the wild type strain we used for this study, is expressed at the stationary phase, not at the exponential phase [20]. Another sRNA FasX influences the expression of fibronectin-binding adhesin, fibrinogen-binding protein, and streptokinase, which converts plasminogen to blood clot-dissolving protease, plasmin. FasX appears to be the main effector molecule of the Fas (fibronectin/fibrinogen binding/hemolytic activity/streptokinase regulator) operon that consists of genes encoding two putative histidine kinases (FasB and FasC) and one response regulator (FasA, SPyM3_0174) [12]. Ramirez-Pena and coworkers revealed the mechanism by which FasX controls the expression of streptokinase; FasX increases the stability of streptokinase mRNA by binding the 5′ end of the mRNA [44]. RivX is an sRNA located downstream of the transcriptional regulator RivR. RivR and RivX activate the Mga regulon composed of genes involved in initial colonization and immune evasion and are repressed directly by CovR, the response regulator of the CovRS (or CsrRS) two-component system [15]. The 4.5S RNA, which is an RNA in the ribonucleoprotein complex of the signal recognition particle (SRP), was shown to influence the production of several secreted proteins and is required for the virulence of *S. pyogenes* [16]. Mutation of 4.5S RNA leads the reduction of streptolysin O, NAD-glycohydrolase at the transcriptional level and the cysteine protease SpeB at the post-transcriptional level. Recently, Deltcheva *et al.* discovered that the maturation of crRNAs (CRISPR RNAs; clustered, regularly interspaced short palindromic repeats RNAs) in *S. pyogenes* is performed by a *trans*-encoded small RNA, tracrRNA, with the assistance of RNase III and the CRISPR-associated Dsn1 protein [19]. These examples above clearly show that small non-coding RNAs affect the physiology and pathogenesis of *S. pyogenes*.

*S. pyogenes* genes are differentially expressed during growth phases (reviewed in [45]). Generally, the factors necessary for colonization such as adhesins and immune evasion factors are expressed more at the exponential phase and the factors involved in persistence and spread are more expressed at the stationary phase. This growth phase-dependent differential expression may be triggered by nutritional status, quorum sensing, cell cycle status, metabolic by-products, pH or other factors involved in or derived from each growth phase. Many sRNAs are key components of regulatory cascades managing environmental change [46]. Intracellular abundance of most novel sRNAs varied between different growth phases (Figure 4), so some of the sRNAs may be involved in coordinating the expression of genes in response to environmental or other signals derived from growth phases.

The majority of *trans*-acting sRNA regulates translation by binding to mRNAs, so identification of target mRNAs of an sRNA would help define the role of the sRNA. Two approaches have been employed to predict target mRNAs of an sRNA: computational prediction approach using bioinformatics and experimental approaches such as genomics- or proteomics-based approaches. The bioinformatic approach is easily accessible since there are several algorithms available online such as TargetRNA [47] (http://snowwhite.wellesley.edu/targetRNA/), RNApredator [48] (http://rna.tbi.univie.ac.at/RNApredator2/target_search.cgi) and IntaRNA [49] (http://rna.informatik.uni-freiburg.de:8080/v1/IntaRNA.jsp). Generally, these algorithms have been developed on the basis of the information of antisense-target RNA interactions previously identified experimentally. When we used these algorithms to find target mRNAs of the new sRNAs, there was not much overlap of target mRNA candidates between these algorithms. The general experimental approaches of high throughput screening for identification of target mRNAs are genomics- and proteomics-based approaches [50]. Between these experimental approaches, a genomics approach using microarray has been preferred. Generally, sRNAs affect translation, not transcription. However, influence of translation of mRNAs appears to influence mRNA degradation by RNases. Thus, microarray-based approaches that compare transcriptional profiles between a pair of strains of an sRNA null mutant and the wild type strain or an over-expressing mutant have been used successfully to identify sRNA targets [51], [52], [53], [54], [55], [56], [57], [58]. Degradation of some target RNAs may not be influenced by binding their cognate sRNAs in some cases. In this case, a proteomics-based approach based on 2-D gel electrophoresis would be useful to identify the targets of sRNAs. However, a proteomics-based approach has a downside of limited coverage of proteins. The expression levels of proteins produced by cells cover a wide range and many of them are not abundant. In many cases, sRNA regulates the expression of regulatory proteins, whose expression level is much lower than that of housekeeping proteins. Because of this limitation, the proteomics-based approach has not been used widely. Also, in *S. pyogenes,* many important proteins in physiology and pathogenesis are tightly associated with the cell wall, which makes the use of proteomics-based approaches more difficult.

**Figure 5:**
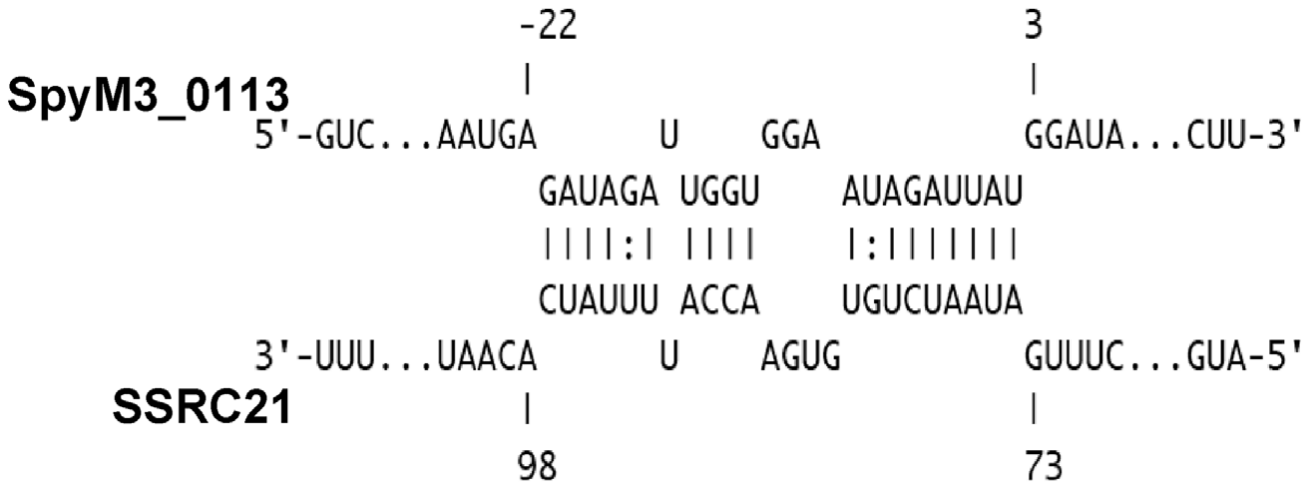
Computational prediction of an interaction between the transcript of a putative regulator SpyM3_0113 and SSRC21. The drawing was generated with the algorithm IntaRNA [49].

To screen target mRNAs of a trans-acting sRNA, we used a genomics-based approach employing the next generation sequencing, RNA-Seq. We chose SSRC21 for this analysis because i) it has been identified by previous studies {Patenge, 2012 #115;Perez, 2009 #71}, ii) its sequence analysis after circular RACE provided predictable promoter and Rho-independent transcription terminator, iii) its differential expression might imply differential regulation of transcripts linked to growth phases, and iv) The possibility that SSRC21 controls the expression of several virulence factors was suggested by computational prediction. The differential RNA sequencing showed that SSRC21 influences transcript abundance of 142 genes (Table S7). Among those, all the 8 *ntp* genes encoding V-type ATPase subunits were notably more abundant in the SSRC21 mutant. It seems that the *ntp* genes are in an operon, so SSRC21 might interact with the *ntp* genes transcript or the transcript of a transcriptional regulator. A transcript encoding a putative regulator upstream of the *ntp* genes was also more abundant in the SSRC21. All of the computational algorithms mentioned above predicted an interaction between the transcript of the regulator and SSRC21 (Figure 5). The role of the V-type ATPase in the physiology and pathogenesis of *S. pyogenes* is not known. In eukaryotes, V-type ATPase is located in organelle membranes and pumps hydrogen ion (H^+^) from the cytosol to the organelles such as golgi and lysosome to acidify the inside of them [59]. Thus, the V-type ATPase in *S. pyogenes* might be involved in pumping hydrogen ion from the cytosol to overcome acid stress during growth or infection. *S. pyogenes* performs only lactic acid fermentation for production of energy and lowers the pH to ∼5.4 in THY medium. In addition, *S. pyogenes* should survive the acidic condition inside the host's lysosome for successful infection. Another possible role of the V-type ATPase in *S. pyogenes* is to balance sodium ion concentration in the cytosol. *Enterococcus hirae* has a homologous V-type Na^+^ ATPase complex that pumps Na^+^ at high pH [60]. The V-type Na+ ATPase confers *E. hirae* the ability to grow at pH 9.5. However, *S. pyogenes* cannot grow at pH 9.5. Several transcripts encoding virulence factors showed differential abundance in the SSRC21 mutant over the wild type. Among them, the two virulence genes encoding M protein and C5A peptidase are in an operon. Since their expression levels were similar, SSRC21 might control the translation of Mga, the transcriptional regulator for M protein and C5A peptidase. However, computational prediction did not detect any significant interaction between SSRC21 and the *mga* transcript or the M protein transcript, so the influence of SSRC21 on the expression of M protein and C5A peptidase might be indirect.

In summary, we searched for small regulatory RNAs in the human pathogen *S. pyogenes* and identified 7 novel streptococcal sRNAs. Since their abundance varied between growth phases, these new sRNAs may coordinate the expression of genes in response to stress conditions linked to growth phases. Differential RNA sequencing analysis to screen putative target mRNAs of an sRNA implied that SSRC21 might be involved in the tolerance to acid stress during growth and/or infection or in the homeostasis of sodium ion inside cells. The list and expression pattern of the novel sRNAs discovered in this study provide a significant resource for future study of small RNAs and their role in *S. pyogenes*.

## Supporting Information

Table S1 **The number of** ***S. pyogenes*** **MGAS315 IGRs that have homologous sequences in the selected** ***Streptococcus*** **genome.**
(DOCX)

Table S2 **Calculation of sensitivity and specificity of RNAz and eQRNA independently and of the combination of the two analyses using known** ***S. pyogenes*** **RNAs.**
(DOCX)

Table S3 **Northern blot probe sequences.**
(DOCX)

Table S4 **RT-PCR and real time RT-PCR primer sequences.**
(DOCX)

Table S5 **Primers used in circular RACE.**
(DOCX)

Table S6 **The nucleotide coordinates of** ***S. pyogenes*** **Small RNA Candidates (SSRC) predicted by each computational algorithm.**
(DOCX)

Table S7 **Differential abundance of transcripts in the SSRC21 deletion mutant, compared to the wild type.**
(DOCX)
