# Supplementary material for: Novel Regulatory Small RNAs in Streptococcus pyogenes
Source: PLoS One. 2013 Jun 6;8(6):e64021. doi: 10.1371/journal.pone.0064021 (PMC3675131; doi:10.1371/journal.pone.0064021)
Supplement: Table S1 — The number of S. pyogenes MGAS315 IGRs that have homologous sequences in the selected Streptococcus genome. (DOCX) [file pone.0064021.s001.docx]

Table S1. The number of *S. pyogenes* MGAS315 IGRs that have homologous sequences in the selected *Streptococcus* genome

| *Streptococcus* genome (Subject^a^) | Number^b^ of *S. pyogenes* IGRs (Query) that showed up in the BLASTN alignments | tRNAs and tmRNA of *S. pyogenes* (Query, total 68) that showed up in the BLASTN alignments |
| --- | --- | --- |
| *S. equi* *zooepidemicus* MGCS 10565 | 337 | 67 |
| *S. mutans* UA159 | 143 | 68 |
| *S. suis* 05ZYH33 | 101 | 65 |
| *S. sanguinis* SK36 | 93 | 66 |
| *S*. *gordonii str. Challis substr.* CH1 | 92 | 66 |
| *S. pneumoniae* CGSP14 | 96 | 66 |
| *S. agalactiae* NEM316 | 197 | 68 |

^a^ Subject is each genome sequence of the seven selected streptococcal species. Each number in the table represents the number of pairwise alignments after query sequences interrogated each subject species.

^b^ Redundant IGRs were removed, so each IGR represents a unique locus.
