# Supplementary material for: Novel Regulatory Small RNAs in Streptococcus pyogenes
Source: PLoS One. 2013 Jun 6;8(6):e64021. doi: 10.1371/journal.pone.0064021 (PMC3675131; doi:10.1371/journal.pone.0064021)
Supplement: Table S2 — Calculation of sensitivity and specificity of RNAz and eQRNA independently and of the combination of the two analyses using known S. pyogenes RNAs. (DOCX) [file pone.0064021.s002.docx]

Table S2. Calculation of sensitivity and specificity of RNAz and eQRNA independently and of the combination of the two analyses using known *S. pyogenes* RNAs

|  | BLASTN^a^ | eQRNA^b^ | RNAz^b^ | Overlap between QRNA and RNAz^b^ |
| --- | --- | --- | --- | --- |
| Real alignments^c^ | 68/68 | 66/68 | 65/68 | 65/68 |
| Shuffled alignments^d^ | 68/68 | 16/68 | 3/68 | 1/68 |

^a^ BLAST comparisons detected 68 of the 68 known RNAs tested.

^b^ To estimate the specificity of both methods combined, both programs scored identical shuffled windows.

^c^ 67 tRNAs and 1 tmRNA were used in order to get the sensitivity.

^d^ Shuffled 67 tRNAs and 1 tmRNA were used to score the number of false positive alignments, which represents the specificity of our approach.
