# Supplementary material for: Novel Regulatory Small RNAs in Streptococcus pyogenes
Source: PLoS One. 2013 Jun 6;8(6):e64021. doi: 10.1371/journal.pone.0064021 (PMC3675131; doi:10.1371/journal.pone.0064021)
Supplement: Table S3 — Northern blot probe sequences. (DOCX) [file pone.0064021.s003.docx]

Table S3. Northern blot probe sequences

| **5’Probe** | **Sequence** | **3’Probe** | **Sequence** |
| --- | --- | --- | --- |
| RNA ladder | TAGGGAAGAGCATTGTCGAG |  |  |
| 5SSRC1 | CACTAGTTTCACTAAACTGAACCCGAGCGGAAAGCTCGGA | 3SSRC1 | TTAGGAAACCAACAATGGACGCTTGCGTCCTAGGCGGTTT |
| 5SSRC2 | TAAGAGCGTCGTGGACAAAGTGAAAATAGGAAATCTGACG | 3SSRC2 | AACACGAGCTGTGGTCTGTGTGAAAAAGATAAATCTTCCT |
| FasX (SSRC4) | CAAACAAAGACAACTGACATCGTCATTTTGTCTGCCGAGC |  |  |
| 5SSRC5 | AGGGAAATTGACGCAGAAACTTCAGTTTCTAGGCAATTTT | 3SSRC5 | CTCAGGCTAAACGCTGTGTGAAAAAGATAAACTTTCCTAG |
| 5SSRC6 | AAAGGCTAAGATGGAAGGGGTGTCTTAGCCTTTTTTTGTC | 3SSRC6 | TTCACGAGTACAAACGTTACGGTAAAAAATAAGATTTTAC |
| 5SSRC7 | TTTTGTCTAACGTTTGGGGTGTAGTTCAAGGCTTTTCCTC | 3SSRC7 | TTTTATTTGCCCGCGTTGTAAAATGAAGTGCAACAAAAAAAG |
| 5SSRC8 | CGCCAATCGAGATTTTTGAGACCCTAGGCTCAAAAATAAG | 3SSRC8 | TTCTTAGGTTGTGCTCACGGCAGCGACTTCCTTTGGAATTC |
| 5SSRC9 | AAAAAGATTGCAAAACACTATATCTTGTGTTATTATGGTT | 3SSRC9 | CGCTCTAGTGTCCTCTTTTGTTTGGCTAAAGTTAAGCATGC |
| 5SSRC10 | ATCAAGGGAGTAGCAGACGGCTAGCCGTGATTGTATCGTC | 3SSRC10 | TCGCTGTTTAAGATTGTACCGGAAACCGAAATTCCGTATTG |
| 5SSRC11 | TAGCGACCCTCAAAAGTTGGACCTCTTATGTTATTCCAAGG | 3SSRC11 | TTTGGGGTGCTTTTCTTCTCTTGAAACCTTAGAAACTGCT |
| Pel/*sagA* (SSRC12) | CAATTGAGAAGCAACAAGTAGTACAGCAGCAACAGCAGCC |  |  |
| 5SSRC13 | GGAATTCACTCAGCTAAAGACAATTAATTAAGGTCTGATAAC | 3SSRC13 | ACTTCATAAGCGGAGCGTTGACACGCGGTACCATCCGTTT |
| 5SSRC14 | AGACGGATTAAAAACATGCACTATGATACTTAATTGTGCT | 3SSRC14 | CCTTGGAAACAAGGAGATTCAGCAAAATATAGCACAATTAAG |
| 5SSRC15 | AAAAAGCTGGATACTCTATTTTAAGTAAAGGATTTAGTTC | 3SSRC15 | AACGGCATTGATTATTACAACGATAAACATATCAAAGTAA |
| 5SSRC16 | GAGCCCCAAAAGTTGGCTACTCTATTTGAAGTAAAGGATT | 3SSRC16 | CGTATCAAAATAAAACGAAAAGGACTTAGCCCTCTACAAC |
| 5SSRC17 | TTTGAATTCGGGCTAAGGACTTGGTCAAAAAGATAAGTTG | 3SSRC17 | TGGCAAAATAGGAAGTTGGCAGAAAATCCTGATTTTCTAG |
| 5SSRC18 | AATTGAATCCGAGCGAAAAGCTCGGATAAAAATGGTAAGAG | 3SSRC18 | AAAGCGATTGAATTTTTGCGGTAAGTCAGAAATCTTTGAT |
| 5SSRC19 | CCGTATCAAAATAAAACGAAAAGGACTTAGTCCTCTACAA | 3SSRC19 | GCCCCAAAAGTTGGCTACTCTATTTGAAGTAAAGGATTTAG |
| 5SSRC20 | AAAGTTTTGCCAAATTCATCGTTATTTTTTCTAAAATTTG | 3SSRC20 | GGCATAAAGCCGATACTATTATATCAAATTTTAGAAAAAAT |
| 5SSRC21 | TCAATCCAAGGGGCATTCTTAATAAATAAGATTGTGATAA | 3SSRC21 | AGCCGACTTTGATAATCTGTGTGAACCATTTTATCACAATC |
| 5SSRC22 | CATAACTTACTAAAACCTTGTTACATCAAGGTTTTTTCTT | 3SSRC22 | TCATGAACAAGGCAAAAAGAAAAAACCTTGATGTAACAAGG |
| 5SSRC23 | TGCTATACTAAAACAAGCACAAGGTAAGTACTTCTAAAAAG | 3SSRC23 | TGGAGCCTAGTTCCACTCTTCATAAGGTAAGTGGAAAGAAC |
| 5SSRC24 | TCTTGAATGGGTAAATCCACTGTAGAGATTATAGAGCTTT | 3SSRC24 | AATGAGAGTTTGGTTGCTTTTCATTATAAATCTTATGGGAC |
| 5SSRC25 | AAGAACCTTGTCGCTATCAATAAACATCGATAGCAACAAG | 3SSRC25 | TTAAAAGACGCTGTTAAATAATTCGTCTAGAAAAATCTTG |
| 5SSRC26 | CCCTCTTTGCTCGTAAGGTGAACAAACCTCCTCGCTTACTG | 3SSRC26 | ACAGCAAGCGGGAGATAATGAGAGTCTCGTATTCCCTAAG |
| 5SSRC27 | GAGGTCTTAACTGTCACTACCAAGCATCTCTTTTTAGTTGC | 3SSRC27 | TGAAAGAAAGCTTTTAGCCGATTTTCATTGAGTGTGCAAC |
| 5SSRC28 | TCAAAGCTAAAAGAAAAGGCTAATTCTCAATAAAATTAGC | 3SSRC28 | TTAAAGGAAAAGAGTGTGCCATAATAAAAAACAATAGTAG |
| 5SSRC29 | GCTTACACTGGACAGGGACGAATTTCGTGTTACGATTTCCG | 3SSRC29 | GTCAAGGCTGGTAGCTGAGGATCAGAAATCAGATATGAAA |
| 5SSRC30 | GCCTGTCGTTCAATTATTTCGTCTAAAAAGCCTTAATGAG | 3SSRC30 | AAGCTTGGCTTTCTCATCTCCAGATTAAGCCCTTTAGCTTG |
| 5SSRC31 | TGTGGAGTGAGCCAAAACAGATGTTTTTCGTTGTGGTAAA | 3SSRC31 | GTGCTTAAGGCAGCGACTTCCTTTGGAATTCCATGCCTTA |
| 5SSRC32 | AACTATCCCATGCCCCTACC | 3SSRC32 | CTGAAAGTGCCACAGTGACG |
| 5SSRC33 | TGGTTATTATCCAGCCATTCTATTCTAAAGGATTACTTCT | 3SSRC33 | TCTTGAAGAAAATCAAATAAGGTCTATAATAGAAGTAATCC |
| 5SSRC34 | TGGTGGTTTTTCTTCTGTCAACAGCTATCAAAAAATGAAA | 3SSRC34 | TTGATATTAAAGGTGTTTTCAAGCTGTTTAAAACAAAAATATGC |
| 5SSRC35 | CTTTCGTTTAGCACTCTATTTAGTAGACTGCTAATTTATA | 3SSRC35 | TTTTTCTTGACATGTCTCCCATAAAGAGTATAATAGAATT |
| 5SSRC36 | TTTTCAGGACAACTTTTGATCCTTCCACAAAAACCATGGTG | 3SSRC36 | ATCAACGATTGGAACTAATTTATGGTTCGCACCATGGTTT |
| 5SSRC37 | TCCTATTTCTGCATGTGGGGTGAGCCAAAACAGCTGCTTT | 3SSRC37 | GATCTCAAAAATCCGTATGGGTATCACTGTCAATATTGATG |
| 5SSRC38 | TGTATGATAAAATCACAGCAACTGAAGAGATAAGCAAAAGG | 3SSRC38 | TGTGCTGCCTCATCAAAAAGAAGTTAGGACAAATAGCTGTC |
| 5SSRC40 | CTCATGTTCATCGAGTGCTAACTTTATATTCTATTTTATC | 3SSRC40 | TTTTCTTGACTATTTTTGACCAAGTGATAAAATAGAATAT |
| 5SSRC41 | CCTTCTTTCTAAAATTAAGTGCTATGCTTTATTATAGCTT | 3SSRC41 | CCTTCTTCGCTTGAAATTCTTGTTTATTTAATAGAAATAAGC |
| 5SSRC42 | AGCCCTGTTATCATTTGGATGGCAGGGCTTTTTTATATTTGG | 3SSRC42 | TTAAACTTTGTAGATGTCAATTTCAGCTACACCAAAATATAG |
| 5SSRC43 | AACAGCTAAAATAGAAAATACTTAACCTTTACAGAAGTAA | 3SSRC43 | GAAACGTTCATTTCATAGGCTTTTCTCATCTCAATTATTTTAC |
| 5SSRC44 | AACCTCTTAAGCACACATTATACCATAAAAAAGCCCTGAA | 3SSRC44 | AAATGAAATTGTAATCTCAAAGCAAGTGGAAGCTCTGTAT |
| 5SSRC45 | TCTTCTTCTTAGCGACATTAAAAAGTGAGATAATATCATT | 3SSRC45 | TTTGAGGAATTGAAAACCGCTATCTCAACTATATTAATCC |
