# Supplementary material for: Novel Regulatory Small RNAs in Streptococcus pyogenes
Source: PLoS One. 2013 Jun 6;8(6):e64021. doi: 10.1371/journal.pone.0064021 (PMC3675131; doi:10.1371/journal.pone.0064021)
Supplement: Table S4 — RT-PCR and real time RT-PCR primer sequences. (DOCX) [file pone.0064021.s004.docx]

Table S4. RT-PCR and real time RT-PCR primer sequences

| **5’Primer** | **Sequence** | **3’Primer** | **Sequence** |
| --- | --- | --- | --- |
| 5SSR1 | CACTAAACTGAACCCGAGCG | 3SSR1 | TTAGGAAACCAACAATGGACG |
| 5SSR2 | TAAGAGCGTCGTGGACAAAG | 3SSR2 | AACACGAGCTGTGGTCTGTG |
| 5SSR3 | GCGATAATAAAGTGCGCTTCC or ATAATAAAGTGCGCTTCCAG | 3SSR3 | TCCTCTTGTAGGATCTTCTTCCC or CTCTTGTAGGATCTTCTTCCC |
| 5SSR4 | GATATGATGGCTCGGCAGAC | 3SSR4 | CCGGGCTTTAATACTGATTTTAC |
| 5SSR5 | AGGGAAATTGACGCAGAAAC | 3SSR5 | CTCAGGCTAAACGCTGTGTG |
| 5SSR6 | AAAGGCTAAGATGGAAGGGG | 3SSR6 | TTCACGAGTACAAACGTTACGG |
| 5SSR7 | TTTTGTCTAACGTTTGGGGTG | 3SSR7 | TTTTATTTGCCCGCGTTG |
| 5SSR8 | CGCCAATCGAGATTTTTGAG | 3SSR8 | TTCTTAGGTTGTGCTCACGG |
| 5SSR9 | AAAAAGATTGCAAAACACTATATC | 3SSR9 | CGCTCTAGTGTCCTCTTTTG |
| 5SSR10 | ATCAAGGGAGTAGCAGACGG | 3SSR10 | TCGCTGTTTAAGATTGTACCG |
| 5SSR11 | TAGCGACCCTCAAAAGTTGG | 3SSR11 | TTTGGGGTGCTTTTCTTCTC |
| 5SSR12 | AGATGAATGGCATAGAGGTGTTAG | 3SSR12 | ACCTGCTAATTACCTGAGACGC |
| 5SSR13 | GGAATTCACTCAGCTAAAGACAA | 3SSR13 | ACTTCATAAGCGGAGCGTTG |
| 5SSR14 | AGACGGATTAAAAACATGCACTATG | 3SSR14 | CCTTGGAAACAAGGAGATTCAG |
| 5SSR15 | AAAAAGCTGGATACTCTATTTTAAG | 3SSR15 | AACGGCATTGATTATTACAAC |
| 5SSR16 | GAGCCCCAAAAGTTGGCTAC | 3SSR16 | CGTATCAAAATAAAACGAAAAGGAC |
| 5SSR17 | TTTGAATTCGGGCTAAGGAC | 3SSR17 | TGGCAAAATAGGAAGTTGGC |
| 5SSR18 | AATTGAATCCGAGCGAAAAG | 3SSR18 | AAAGCGATTGAATTTTTGCG |
| 5SSR19 | CCGTATCAAAATAAAACGAAAAGG | 3SSR19 | GCCCCAAAAGTTGGCTACTC |
| 5SSR20 | AAAGTTTTGCCAAATTCATC | 3SSR20 | GGCATAAAGCCGATACTATT |
| 5SSR21 | TCAATCCAAGGGGCATTC | 3SSR21 | AGCCGACTTTGATAATCTGTGTG |
| 5SSR22 | CATAACTTACTAAAACCTTGTTACATC | 3SSR22 | TCATGAACAAGGCAAAAAG |
| 5SSR23 | TGCTATACTAAAACAAGCACAAGG | 3SSR23 | TGGAGCCTAGTTCCACTCTTC |
| 5SSR24 | TCTTGAATGGGTAAATCCACTG | 3SSR24 | AATGAGAGTTTGGTTGCTTTTC |
| 5SSR25 | AAGAACCTTGTCGCTATCAATAAAC | 3SSR25 | TTAAAAGACGCTGTTAAATAATTCG |
| 5SSR26 | CCCTCTTTGCTCGTAAGGTG | 3SSR26 | ACAGCAAGCGGGAGATAATG |
| 5SSR27 | GAGGTCTTAACTGTCACTACCAAGC | 3SSR27 | TGAAAGAAAGCTTTTAGCCG |
| 5SSR28 | TCAAAGCTAAAAGAAAAGGC | 3SSR28 | TTAAAGGAAAAGAGTGTGCC |
| 5SSR29 | GCTTACACTGGACAGGGACG | 3SSR29 | GTCAAGGCTGGTAGCTGAGG |
| 5SSR30 | GCCTGTCGTTCAATTATTTCG | 3SSR30 | AAGCTTGGCTTTCTCATCTCC |
| 5SSR31 | TGTGGAGTGAGCCAAAACAG | 3SSR31 | GTGCTTAAGGCAGCGACTTC |
| 5SSR32 | AACTATCCCATGCCCCTACC | 3SSR32 | CTGAAAGTGCCACAGTGACG |
| 5SSR33 | TGGTTATTATCCAGCCATTC | 3SSR33 | TCTTGAAGAAAATCAAATAAGG |
| 5SSR34 | TGGTGGTTTTTCTTCTGTCAAC | 3SSR34 | TTGATATTAAAGGTGTTTTCAAGCTG |
| 5SSR35 | CTTTCGTTTAGCACTCTATTTAGTA | 3SSR35 | TTTTTCTTGACATGTCTCCC |
| 5SSR36 | TTTTCAGGACAACTTTTGATCCTTC | 3SSR36 | ATCAACGATTGGAACTAATTTATGG |
| 5SSR37 | TCCTATTTCTGCATGTGGGG | 3SSR37 | GATCTCAAAAATCCGTATGGG |
| 5SSR38 | TGTATGATAAAATCACAGCAACTG | 3SSR38 | TGTGCTGCCTCATCAAAAAG |
| 5SSR39 | AGCTGTCCATCTGTGCGTAG | 3SSR39 | CATCAAGAGAGAACCAGTCGG |
| 5SSR40 | CTCATGTTCATCGAGTGCTAAC | 3SSR40 | TTTTCTTGACTATTTTTGACCAAGTG |
| 5SSR41 | CCTTCTTTCTAAAATTAAGTGCTATGC | 3SSR41 | CCTTCTTCGCTTGAAATTCTTG |
| 5SSR42 | AGCCCTGTTATCATTTGGATG | 3SSR42 | TTAAACTTTGTAGATGTCAATTTCAGC |
| 5SSR43 | AACAGCTAAAATAGAAAATACTTAACC | 3SSR43 | GAAACGTTCATTTCATAGGC |
| 5SSR44 | AACCTCTTAAGCACACATTATACC | 3SSR44 | AAATGAAATTGTAATCTCAAAGC |
| 5SSR45 | TCTTCTTAGCGACATTAAAAAGTGAG | 3SSR45 | TTTGAGGAATTGAAAACCGC |
