# Supplementary material for: Novel Regulatory Small RNAs in Streptococcus pyogenes
Source: PLoS One. 2013 Jun 6;8(6):e64021. doi: 10.1371/journal.pone.0064021 (PMC3675131; doi:10.1371/journal.pone.0064021)
Supplement: Table S5 — Primers used in circular RACE. (DOCX) [file pone.0064021.s005.docx]

Table S5. Primers used in circular RACE

| **5’ Primer** | **Sequence** | **3’ Primer** | **Sequence** |
| --- | --- | --- | --- |
| 5SSRC8 | CCAATCGAGATTTTTGAGACC | 3SSRC8 | TTTCCATAGCAAACAGCAGC |
| 5SSRC10 | AATACGGAATTTCGGTTTCC | 3SSRC10 | ACGATACAATCACGGCTAGC |
| 5SSRC21 | TGTGTGAACCATTTTATCACAATC | 3SSRC21 | TTATCAAAGTCGGCTTCCC |
| 5SSRC29 | AAAAGTAGCAGATCCATTTTATGG | 3SSRC29 | CATATCTGATTTCTGATCCTCAGC |
| 5SSRC34 | TTGACAGAAGAAAAACCACC | 3SSRC34 | CAGCTATCAAAAAATGAAAATC |
| 5SSRC41 | GAAGGTATTTAAAAAAAGCTCTACAAG | 3SSRC41 | CGCTTGAAATTCTTGTTTATTTAATAG |

The 3’ primers were used to synthesize first strand cDNA and both 5’ and 3’ primers were used to amplify the first strand cDNA through PCR.
