# Supplementary material for: Novel Regulatory Small RNAs in Streptococcus pyogenes
Source: PLoS One. 2013 Jun 6;8(6):e64021. doi: 10.1371/journal.pone.0064021 (PMC3675131; doi:10.1371/journal.pone.0064021)
Supplement: Table S6 — The nucleotide coordinates of S. pyogenes Small RNA Candidates (SSRC) predicted by each computational algorithm. (DOCX) [file pone.0064021.s006.docx]

Table S6. The nucleotide coordinates of *S. pyogenes* Small RNA Candidates (SSRC) predicted by each computational algorithm.

| SSRC # | Algorithm | Start^a^ | End^a^ |
| --- | --- | --- | --- |
| SSRC1 | eQRNA | 51887 | 52049 |
|  | RNAz | 51897 | 52041 |
| SSRC2 | eQRNA | 71903 | 72048 |
|  | RNAz | 71903 | 72016 |
| SSRC3 | eQRNA | 186137 | 186254 |
|  | RNAz | 186136 | 186257 |
| SSRC4 | eQRNA | 205345 | 205632 |
|  | RNAz | 205479 | 205629 |
| SSRC5 | eQRNA | 228481 | 228853 |
|  | RNAz | 228481 | 228848 |
| SSRC6 | eQRNA | 232542 | 232821 |
|  | RNAz | 232542 | 232750 |
| SSRC7 | eQRNA | 255844 | 256290 |
|  | sRNAPredict | 255793 | 255940 |
| SSRC8 | eQRNA | 339573 | 339720 |
|  | RNAz | 339554 | 339703 |
|  | sRNAPredict | 339684 | 339715 |
| SSRC9 | eQRNA | 343442 | 343574 |
|  | RNAz | 343442 | 343574 |
| SSRC10 | eQRNA | 482014 | 482212 |
|  | sRNAPredict | 482013 | 482202 |
| SSRC11 | eQRNA | 501033 | 501168 |
|  | RNAz | 500822 | 501050 |
| SSRC12 | eQRNA | 529799 | 529955 |
|  | RNAz | 529785 | 529944 |
|  | sRNAPredict | 529735 | 529922 |
| SSRC13 | eQRNA | 554924 | 555067 |
|  | RNAz | 555032 | 555165 |
| SSRC14 | eQRNA | 611417 | 611581 |
|  | sRNAPredict | 611476 | 611554 |
| SSRC15 | eQRNA | 641075 | 641322 |
|  | RNAz | 641082 | 641207 |
|  | sRNAPredict | 641118 | 641206 |
| SSRC16 | eQRNA | 648379 | 648715 |
|  | RNAz | 648434 | 648574 |
| SSRC17 | eQRNA | 671784 | 672476 |
|  | RNAz | 672280 | 672426 |
| SSRC18 | eQRNA | 726973 | 727164 |
|  | RNAz | 726973 | 727178 |
| SSRC19 | eQRNA | 728969 | 729393 |
|  | RNAz | 729228 | 729370 |
| SSRC20 | eQRNA | 816084 | 816198 |
|  | sRNAPredict | 816084 | 816207 |
| SSRC21 | eQRNA | 907742 | 907890 |
|  | sRNAPredict | 907889 | 907989 |
| SSRC22 | eQRNA | 977284 | 977393 |
|  | RNAz | 977283 | 977393 |
| SSRC23 | eQRNA | 1025855 | 1026057 |
|  | RNAz | 1025885 | 1026058 |
| SSRC24 | eQRNA | 1029469 | 1029713 |
|  | RNAz | 1029469 | 1029599 |
| SSRC25 | eQRNA | 1137220 | 1137581 |
|  | RNAz | 1137492 | 1137581 |
| SSRC26 | eQRNA | 1194303 | 1194554 |
|  | RNAz | 1194285 | 1194551 |
| SSRC27 | eQRNA | 1204316 | 1204422 |
|  | sRNAPredict | 1204500 | 1204577 |
| SSRC28 | eQRNA | 1216938 | 1217114 |
|  | RNAz | 1216965 | 1217114 |
| SSRC29 | eQRNA | 1282037 | 1282390 |
|  | sRNAPredict | 1282148 | 1282325 |
| SSRC30 | eQRNA | 1285428 | 1285860 |
|  | RNAz | 1285427 | 1285577 |
|  | sRNAPredict | 1285643 | 1285827 |
| SSRC31 | eQRNA | 1352788 | 1353126 |
|  | RNAz | 1352788 | 1353038 |
| SSRC32 | eQRNA | 1388604 | 1389047 |
|  | RNAz | 1388904 | 1389047 |
|  | sRNAPredict | 1388626 | 1388724 |
| SSRC33 | eQRNA | 1394655 | 1394802 |
|  | RNAz | 1394658 | 1394802 |
|  | sRNAPredict | 1394707 | 1394804 |
| SSRC34 | eQRNA | 1503057 | 1503406 |
|  | RNAz | 1503276 | 1503407 |
| SSRC35 | eQRNA | 1525819 | 1525936 |
|  | RNAz | 1525805 | 1525938 |
| SSRC36 | eQRNA | 1654505 | 1654645 |
|  | RNAz | 1654565 | 1654708 |
| SSRC37 | eQRNA | 1683031 | 1683317 |
|  | RNAz | 1683091 | 1683239 |
| SSRC38 | eQRNA | 1742151 | 1742322 |
|  | RNAz | 1742111 | 1742292 |
| SSRC39 | eQRNA | 1745833 | 1746187 |
|  | RNAz | 1745833 | 1746190 |
| SSRC40 | eQRNA | 1787418 | 1787549 |
|  | sRNAPredict | 1787401 | 1787549 |
| SSRC41 | eQRNA | 1828383 | 1828494 |
|  | RNAz | 1828383 | 1828494 |
| SSRC42 | eQRNA | 1848673 | 1848922 |
|  | RNAz | 1848723 | 1848952 |
| SSRC43 | eQRNA | 1852487 | 1852634 |
|  | RNAz | 1852439 | 1852660 |
| SSRC44 | eQRNA | 1865897 | 1865985 |
|  | RNAz | 1865897 | 1865985 |
| SSRC45 | eQRNA | 1873136 | 1873410 |
|  | RNAz | 1873188 | 1873397 |

^a^ Nucleotide coordinates are based on the genome sequence of *S. pyogenes* MGAS315.
