# Supplementary material for: Novel Regulatory Small RNAs in Streptococcus pyogenes
Source: PLoS One. 2013 Jun 6;8(6):e64021. doi: 10.1371/journal.pone.0064021 (PMC3675131; doi:10.1371/journal.pone.0064021)
Supplement: Table S7 — Differential abundance of transcripts in the SSRC21 deletion mutant, compared to the wild type. (DOCX) [file pone.0064021.s007.docx]

Table S7. Differential abundance of transcripts in the SSRC21 deletion mutant, compared to the wild type

| **Gene** | **Putative function** | **Fold change** |
| --- | --- | --- |
| *adh1, SpyM3_0037* | alcohol dehydrogenase | 2.29 |
| *spyM3_0112* | hypothetical protein | 3.72 |
| *spyM3_0113* | regulatory protein | 4.25 |
| *spyM3_0114* | hypothetical protein | 7.10 |
| *ntpI, spyM3_0115* | produces ATP from ADP in the presence of a proton gradient across the membrane. Subunit I is part of the membrane proton channel. | 7.42 |
| *ntpK, spyM3_0116* | V-type ATP synthase subunit K | 6.42 |
| *ntpE, spyM3_0117* | V-type Na+ -ATPase subunit E | 6.06 |
| *ntpC, spyM3_0118* | V-type Na+ -ATPase subunit C | 6.52 |
| *ntpG, spyM3_0119* | V-type H+-transporting ATPase subunit F | 6.32 |
| *ntpA, spyM3_0120* | NtpA-produces ATP from ADP in the presence of a proton gradient across the membrane; the A subunit is part of the catalytic core of the ATP synthase complex | 6.20 |
| *ntpB, spyM3_0121* | V-type H+-transporting ATPase subunit B | 6.36 |
| *ntpD, spyM3_0122* | V-type ATP synthase subunit D; produces ATP from ADP in the presence of a proton gradient across the membrane; the D subunit is part of the catalytic core of the ATP synthase complex | 5.77 |
| *spyM3_0131* | hypothetical protein | 2.02 |
| *araD, spyM3_0140* | catalyzes the formation of D-xylulose 5-phosphate from L-ribulose 5-phosphate in the L-arabinose and L-ascorbate degradation pathways | 2.04 |
| *spyM3_0366* | ABC transporter ATP-binding protein | 2.54 |
| *spyM3_0367* | hypothetical protein | 2.16 |
| *glmS, spyM3_0910* | glucosamine--fructose-6-phosphate aminotransferase (isomerizing) | 3.35 |
| *spyM3_0972* | hypothetical protein | 2.68 |
| *spyM3_0973* | hypothetical protein | 2.54 |
| *spyM3_0975* | hypothetical protein | 2.65 |
| *spyM3_0989* | hypothetical protein | 2.32 |
| *spyM3_0990* | esterase | 2.44 |
| *spyM3_1195* | hypothetical protein | 2.05 |
| *spyM3_1203* | hypothetical protein | 2.14 |
| *spyM3_1225* | major capsid/head protein - phage associated | 2.01 |
| *spyM3_1229* | minor capsid protein - phage associated | 2.68 |
| *spyM3_1235* | ABC transporter ATP-binding protein - phage associated | 2.19 |
| *spyM3_1258* | hypothetical protein | 2.22 |
| *spyM3_1259* | hypothetical protein | 2.32 |
| *spyM3_1260* | hypothetical protein-phage associated | 2.58 |
| *spyM3_1261* | P1-type antirepressor - phage associated | 2.65 |
| *spyM3_1298* | two-component response regulator histidine kinase | 2.67 |
| *deoC, spyM3_1611* | catalyzes the formation of D-glyceraldehyde 3-phosphate and acetaldehyde from 2-deoxy-D-ribose-5-phosphate | 2.16 |
| *spyM3_1669* | SpoU family tRNA/rRNA methyltransferase | 3.49 |
| *def, spyM3_1684* | cleaves off formyl group from N-terminal methionine residues of newly synthesized proteins; binds iron(2+) | 2.56 |
| *spyM3_1685* | hypothetical protein | 3.28 |
| *lrp, spyM3_1697* | leucine-rich protein | 2.19 |
| *ska, spyM3_1698* | streptokinase A precursor | 2.59 |
| *spyM3_t63* | tRNA-Lys | 2.24 |
| *groES, spyM3_1766* | 10 kDa chaperonin; Cpn10; GroES; forms homoheptameric ring; binds to one or both ends of the GroEL double barrel in the presence of adenine nucleotides capping it; folding of unfolded substrates initiates in a GroEL-substrate bound and capped by GroES; release of the folded substrate is dependent on ATP binding and hydrolysis in the trans ring | 2.44 |
| *clpC, spyM3_1767* | endopeptidase Clp ATP-binding chain C | 2.17 |
| *csp, spyM3_1769* | cold-shock protein | 3.44 |
| *spyM3_r02* | 23S ribosomal RNA | -2.84 |
| *spyM3_r03* | 16S ribosomal RNA | -2.99 |
| *spyM3_r04* | 23S ribosomal RNA | -2.87 |
| *spyM3_0014* | secreted protein - glucan-binding protein B | -2.19 |
| *spyM3_0025* | choline binding protein | -2.04 |
| *spyM3_r05* | 16S ribosomal RNA | -2.72 |
| *spyM3_r06* | 23S ribosomal RNA | -2.98 |
| *spyM3_0077* | DNA binding protein | -2.06 |
| *opuAA, spyM3_0143* | glycine betaine/proline ABC transporter (ATP-binding protein) | -2.26 |
| *opuABC, spyM3_0144* | glycine-betaine binding permease protein | -2.29 |
| *spyM3_r07* | 16S ribosomal RNA | -2.93 |
| *spyM3_r08* | 23S ribosomal RNA | -3.01 |
| *spyM3_r16* | 5S ribosomal RNA | -3.17 |
| *spyM3_t43* | tRNA-Asn | -4.30 |
| *spyM3_0331* | histidine kinase | -2.05 |
| *spyM3_0396* | hypothetical protein | -4.73 |
| *spyM3_0467* | GntR family transcriptional regulator | -2.59 |
| *spyM3_0582* | peptidoglycan hydrolase | -3.04 |
| *spyM3_0689* | hypothetical protein | -4.30 |
| *spyM3_0714* | hypothetical protein-phage associated | -405564409.00 |
| *spyM3_0724* | hypothetical protein | -2.01 |
| *spyM3_0726* | hypothetical protein | -2.06 |
| *spyM3_0732* | hypothetical protein | -2.00 |
| *spyM3_0733* | hypothetical protein | -2.35 |
| *spyM3_0734* | hypothetical protein | -6.59 |
| *spyM3_0735* | hypothetical protein | -3.01 |
| *spyM3_0797* | 4-oxalocrotonate tautomerase | -2.33 |
| *spyM3_0851* | anaerobic ribonucleotide reductase | -2.20 |
| *spyM3_0941* | hypothetical protein | -6.66 |
| *clpP.2, spyM3_0942* | ClpP protease ATP-dependent protease proteolytic subunit - phage associated | -3.33 |
| *spyM3_0943* | hypothetical protein | -2.81 |
| *spyM3_0944* | hypothetical protein-phage associated | -2.26 |
| *spyM3_0945* | hypothetical protein | -2.61 |
| *spyM3_0946* | hypothetical protein | -3.68 |
| *spyM3_0947* | hypothetical protein | -3.42 |
| *spyM3_0948* | hypothetical protein | -2.64 |
| *spyM3_0949* | hypothetical protein | -2.34 |
| *spyM3_0950* | hypothetical protein | -3.27 |
| *spyM3_0951* | hypothetical protein | -2.35 |
| *spyM3_0954* | hypothetical phage protein | -3.13 |
| *spyM3_0955* | hypothetical protein | -3.44 |
| *spyM3_0956* | hypothetical protein | -3.01 |
| *spyM3_0957* | DNA primase - phage associated | -4.87 |
| *spyM3_0958* | DNA primase - phage associated | -4.17 |
| *spyM3_0959* | hypothetical protein | -2.58 |
| *spyM3_0960* | hypothetical protein | -3.05 |
| *spyM3_0963* | hypothetical protein | -2.79 |
| *spyM3_0968* | hypothetical protein | -3.76 |
| *spyM3_1004* | transcriptional antiterminator | -2.78 |
| *spyM3_1055* | hypothetical protein | -2.10 |
| *spyM3_1057* | transcriptional regulator protein; similar to transcriptional repressor | -3.67 |
| *queA, spyM3_1066* | S-adenosylmethionine:tRNA ribosyltransferase-isomerase | -2.18 |
| *recR, spyM3_1085* | recombination protein RecR - involved in a recombinational process of DNA repair, independent of the recBC complex | -2.18 |
| *spyM3_1088* | hypothetical protein | -2.40 |
| *gpmA, spyM3_1090* | phosphoglyceromutase | -2.15 |
| *pyrD, spyM3_1091* | catalyzes the conversion of dihydroorotate to orotate in the pyrimidine biosynthesis pathway; subclass 1A is a dimer formed by two identical PyrD subunits each containing an FMN group | -3.01 |
| *spyM3_1092* | hypothetical protein | -3.01 |
| *spyM3_1094* | hyaluronidase | -2.20 |
| *pblB, spyM3_1102* | platelet-binding protein-phage associated | -4.73 |
| *spyM3_1103* | hypothetical protein | -5.37 |
| *spyM3_1105* | hypothetical protein | -3.12 |
| *spyM3_1124* | hypothetical protein | -2.98 |
| *spyM3_1126* | hypothetical protein | -3.52 |
| *sla, spyM3_1204* | streptococcal phospholipase A2 - phage associated | -4.51 |
| *spyM3_1232* | hypothetical protein | -2.20 |
| *spyM3_1247* | hypothetical protein | -3.78 |
| *spyM3_1312* | minor structural protein - phage associated | -709737716.87 |
| *spyM3_1330* | hypothetical protein | -2.14 |
| *spyM3_1331* | hypothetical protein-similar to protein gp51 | -2.67 |
| *spyM3_1332* | hypothetical protein | -2.64 |
| *spyM3_1333* | hypothetical protein - ABC transporter ATP-binding and permease protein | -2.56 |
| *spyM3_1334* | hypothetical protein | -2.19 |
| *spyM3_1335* | hypothetical protein | -2.40 |
| *spyM3_1336* | hypothetical protein | -2.27 |
| *spyM3_1337* | hypothetical protein | -2.39 |
| *spyM3_1356* | hypothetical protein | -3.29 |
| *spyM3_t50* | tRNA-Glu | -3.71 |
| *spyM3_1399* | ATP-dependent RNA helicase | -2.10 |
| *spyM3_1427* | hypothetical protein | -2.66 |
| *spyM3_1434* | hypothetical protein | -2.56 |
| *spyM3_1435* | terminase large subunit - phage associated | -2.24 |
| *spyM3_1436* | terminase small subunit - phage associated | -2.53 |
| *spyM3_1437* | transcriptional activator - phage associated | -4.07 |
| *spyM3_1438* | hypothetical protein | -3.49 |
| *spyM3_1439* | hypothetical protein | -2.31 |
| *spyM3_1440* | helicase - phage associated | -3.68 |
| *spyM3_1441* | hypothetical protein | -2.20 |
| *ropA, spyM3_1634* | Tig; RopA; peptidyl-prolyl cis/trans isomerase; promotes folding of newly synthesized proteins; binds ribosomal 50S subunit; forms a homodimer | -3.18 |
| *spyM3_1636* | hypothetical protein | -3.52 |
| *salX, spyM3_1648* | ABC transporter ATP-binding protein | -2.00 |
| *scpA, spyM3_1726* | C5A peptidase precursor | -2.05 |
| *emm3, spyM3_1727* | antiphagocytic M protein, type 3 | -2.40 |
| *spyM3_1730* | hypothetical protein | -2.09 |
| *spyM3_1741* | hypothetical protein - similar to amino acids 22-128 of pyrogenic exotoxin B | -2.01 |
| *spyM3_1832* | TetR/AcrR family transcriptional regulator | 2.04 |
| *rpsD, spyM3_1833* | primary rRNA binding protein; nucleates 30S assembly; involved in translational accuracy with proteins S5 and S12; interacts with protein S5; involved in autogeneously regulating ribosomal proteins by binding to pseudoknot structures in the polycistronic mRNA; interacts with transcription complex and functions similar to protein NusA in antitermination | -3.67 |

- Gene names and their putative functions are based on the genome of *S. pyogenes* MGAS315 (http://www.ncbi.nlm.nih.gov/nuccore/AE014074.1).

- Genes corresponding to the transcripts showing differential abundance with statistical significance were listed; fold change in the mutant over the wild type greater than 2 or less than -2, p < 0.01, and false discovery rate, RDR < 5%).
